# Integrated proteomics and scRNA-seq analyses of ovarian cancer reveal molecular subtype-associated cell landscapes and immunotherapy targets

**DOI:** 10.1038/s41416-024-02894-2

**Published:** 2024-11-15

**Authors:** Rong Tan, Ming Wen, Wenqing Yang, Dongdong Zhan, Nairen Zheng, Mingwei Liu, Fang Zhu, Xiaodan Chen, Meng Wang, Siyu Yang, Bin Xie, Qiongqiong He, Kai Yuan, Lunquan Sun, Yi Wang, Jun Qin, Yu Zhang

**Affiliations:** 1 Department of Gynecology, Xiangya Hospital, Central South University, Changsha, Hunan China; 2 Xiangya Cancer Center, Xiangya Hospital, Central South University, Changsha, China; 3 Key Laboratory of Molecular Radiation Oncology Hunan Province, Changsha, China; 4 National Clinical Research Center for Geriatric Disorders, Xiangya Hospital, Central South University, Changsha, China; 5 Hunan key laboratory of aging biology, Xiangya Hospital, Central South University, Changsha, China; 6 Gynecological Oncology Research and Engineering Center of Hunan Province, Changsha, Hunan China; 7 State Key Laboratory of Proteomics, Beijing Proteome Research Center, National Center for Protein Sciences (Beijing), Beijing Institute of Lifeomics, Beijing, China; 8 Beijing Pineal Diagnostics Co., Ltd., Beijing, China; 9 Department of Pathology, Xiangya Hospital, Central South University, Changsha, Hunan China; 10 Department of Pathology, School of Basic Medicine, Central South University, Changsha, Hunan China; 11 Hunan International Science and Technology Collaboration Base of Precision Medicine for Cancer, Changsha, China; 12 Center for Molecular Imaging of Central South University, Xiangya Hospital, Changsha, China; 13 State Key Laboratory of Genetic Engineering and Collaborative Innovation Center for Genetics and Development, School of Life Sciences, Institute of Biomedical Sciences, Fudan University, Shanghai, China

**Keywords:** Ovarian cancer, Proteome informatics, Cancer immunotherapy, Targeted therapies, Target identification

## Abstract

**Background:**

Epithelial ovarian cancer (EOC) represents the most lethal gynaecological malignancy, yet understanding the connections between its molecular subtypes and their therapeutic implications remains incomplete.

**Methods:**

We conducted mass spectrometry-based proteomics analyses of 154 EOC tumour samples and 29 normal fallopian tubes, and single-cell RNA sequencing (scRNA-seq) analyses of an additional eight EOC tumours to classify proteomic subtypes and assess their cellular ecosystems and clinical significance. The efficacy of identified therapeutic targets was evaluated in patient-derived xenograft (PDX) and orthotopic mouse models.

**Results:**

We identified four proteomic subtypes with distinct clinical relevance: malignant proliferative (C1), immune infiltrating (C2), Fallopian-like (C3) and differentiated (C4) subtypes. C2 subtype was characterized by lymphocyte infiltration, notably an increased presence of GZMK CD8+ T cells and phagocytosis-like MRC+ macrophages. Additionally, we identified CD40 as a specific prognostic factor for C2 subtype. The interaction between CD40+ phagocytosis-like macrophages and CD40RL+ IL17R CD4+ T cells was correlated with a favourable prognosis. Finally, we established a druggable landscape for non-immune EOC patients and verified a TYMP inhibitor as a promising therapeutic strategy.

**Conclusions:**

Our study refines the current immune subtype for EOC, highlighting CD40 agonists as promising therapies for C2 subtype patients and targeting TYMP for non-immune patients.

## Background

Epithelial ovarian cancer (EOC), presenting the highest mortality rate among gynaecological malignant tumours, constitutes approximately 85% to 90% of ovarian cancer malignancies. Due to the absence of symptoms in the early stages and a lack of appropriate diagnostic methods, 70% to 80% of patients are diagnosed at an advanced stage, characterized by widespread metastasis in the abdominal cavity. Unfortunately, only 31% of women diagnosed with distant invasive EOC survive for 5 years [1]. The low survival rate of advanced EOC primarily results from its high recurrence rate and eventual development of diffuse dissemination and chemo-resistance.

A multi-platform analysis of stage III-IV high-grade serous carcinomas (HGSCs) performed by The Cancer Genome Atlas (TCGA) identified four molecular subtypes characterized by ‘immunoreactive’, ‘differentiated’, ‘proliferative’, and ‘mesenchymal’ signatures [2]. Additionally, a mass spectrometry-based proteomics analysis by the Clinical Proteomic Tumour Analysis Consortium (CPTAC) revealed classifications highly consistent with transcriptome-based clusters [3]. The CPTAC study also identified a distinct molecular subtype defined as stromal, which is enriched with extracellular proteins. As the extracellular matrix, often lost during mRNA extraction, can be properly preserved in protein preparations, this suggests that proteomics has the capacity to capture more comprehensive signatures associated with the tumour microenvironments. Moreover, recent several single-cell RNA sequencing (scRNA-seq) analyses have unveiled tumour-associated T-cell signatures [4–6], classifying the heterogeneous immune microenvironment of ovarian cancer into immune-infiltrated, immune-excluded, and immune-desert subtypes [5]. Those studies shed light on the tumour microenvironment using different multi-omics analyses. However, bridging the gap between molecular features, cell-based ecosystems, and therapeutic strategies in the clinic remains a formidable challenge. Therefore, there is an increasing need for alternative and more precise molecular signatures to guide therapeutic strategies in the majority of ovarian cancers and improve patient survival rates.

Surgery combined with platinum-based chemotherapy remains the standard of therapy for all types of EOC. However, a significant percentage of patients exhibit primary resistance to chemotherapy or experience relapse shortly after treatment initiation. The status of the p53 gene plays a crucial role in determining ovarian cancer’s sensitivity to platinum-based chemotherapy. Patients with certain TP53 mutations, particularly gain-of-function mutations, often show reduced responsiveness to this treatment [7–9]. While PD-1-targeting immunotherapy has achieved significant success in melanoma, endometrial, lung, and kidney cancers [10], common immunotherapy targets like PD-1 and CTLA-4 have been less effective in ovarian cancer. Phase III randomized trials in ovarian cancer have failed to meet their primary endpoints when these therapies were used as monotherapy or in combination with chemotherapy, bevacizumab, or PARP inhibitors [11, 12]. In ovarian cancer, molecularly targeted therapies include angiogenesis inhibitors like bevacizumab and PARP inhibitors such as olaparib, has been shown to improve progression-free survival (PFS) in patients, particularly those with BRCA mutations (BRCAmut) or homologous recombination deficiency (HRD) [13, 14], which accounts for approximately 20% of all ovarian cancer cases [15]. Therefore, identifying new therapeutic strategies, especially immunotherapy, and selecting appropriate patient populations is crucial for improving treatment outcomes in epithelial ovarian cancer (EOC).

In this study, we initially performed proteomic profiling of 154 EOC tumour samples and 29 normal fallopian tissues. The 154 tumour samples included primary tumour lesions from 22 early-stage patients, and primary and their paired omental and peritoneal metastatic lesions from 44 advanced-stage patients. Moreover, 8 additional primary tumours, representing 4 immune and 4 non-immune subtypes, were analysed by proteome and scRNA-seq. The integrated analysis of proteomics with the scRNA-seq identified CD40 as a possible target that could potentiate the immunotherapy response. Finally, a druggable landscape for the non-immune subtype patients was established, and a TYMP inhibitor TAS102 was validated as a promising chemotherapy agent in the patient-derived xenograft (PDX) and orthotopic mouse models.

## Results

### An integrated EOC proteomic analysis identifies four subtypes covering different histological types

**Fig. 1:**
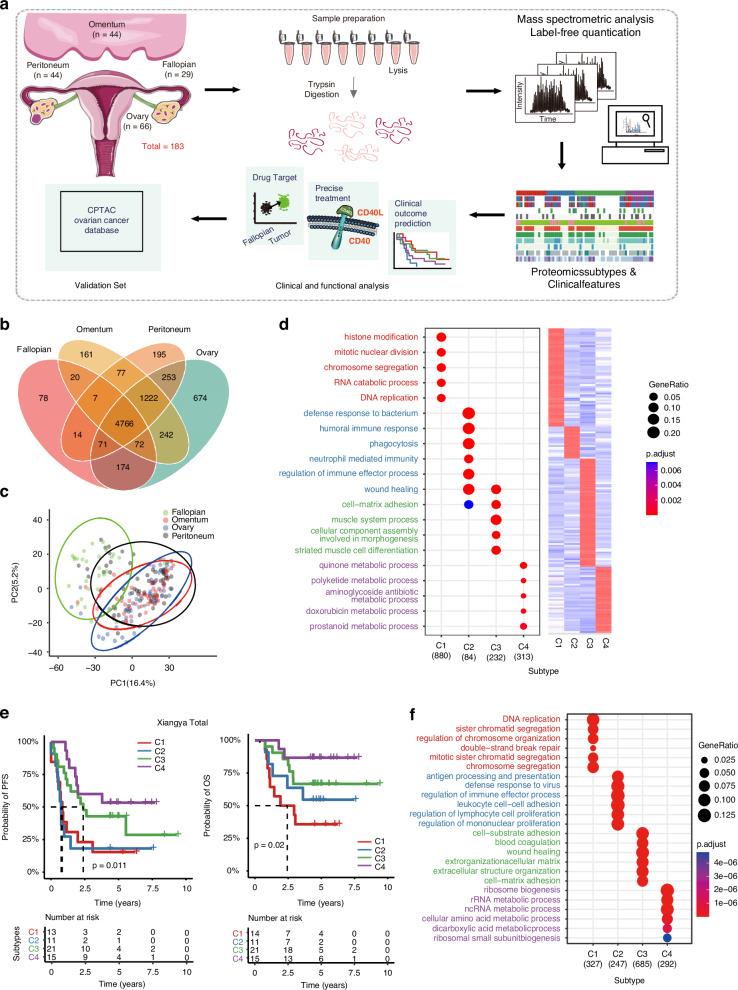
Integrated OC proteomic analysis identifies four Proteomic subtypes associated with prognosis. **a** Workflow diagram for sample collection, MS-based quantitative proteomics, bioinformatic analyses and validations. Following lysis, protein purification, and tryptic digest, peptides were separated by ultra-high performance liquid chromatography and measured in single runs using a quadrupole Orbitrap mass spectrometer. Label-free proteome quantification was performed using the MaxQuant software environment. **b** Venn diagrams depicting overlapped proteins detected in the primary ovary, omentum, peritoneum tumour sites and fallopian tubes. **c** PCA analysis of samples from tissue sites as indicated in Xiangya OC proteomics data. **d** The proteomic average abundance of signature genes for 4 NMF subtyping (right) and the dot plot showing GO-BP (biological progress) analysis of feature proteins referring to Xiangya four Proteomic subtypes (left). **e** The Kaplan-Meier (K-M) curves of progression-free survival (PFS, left) and overall survival (OS, right) for Proteomic subtypes for Xiangya OC patients. The tables below show the numbers of patients in follow-up at the year as indicated. **f** Dotplot showing GO-BP (biological progress) analysis of feature proteins for Proteomic subtypes of 2023 OC validation cohort.

To comprehensively investigate the EOCs covering various pathological statuses, we prospectively collected 183 specimens, including 154 EOC tumour samples and 29 normal fallopian tube tissues. All specimens were obtained from 82 patients before undergoing any treatment. The tumour samples, including 22 primary tumour lesions, 44 primary tumour lesions and their paired omental and peritoneal metastatic lesions) (Fig. S1a, b, detailed sample collection can be found in STAR Methods and Table S1a). All those tumours were acquired through laparoscopy or primary debulking surgeries [16], covering a diverse range of pathological and histological statuses of epithelial ovarian cancer (EOC), including 137 high-grade serous carcinomas (HGSCs) and 17 samples of other histological subtypes (Fig. S1a, b, Table S1a, see Method details). Label-free liquid chromatography (LC)–tandem mass spectrometry (MS/MS) with data-dependent acquisition was conducted, and quantification was performed using iBAQ followed by normalization to iFOT [17–19]. Nonnegative matrix factorization (NMF) analyses [20] were carried out on the 183 specimens for the classification of proteomic subtypes (Fig. 1a). Comprehensive details regarding sample information and clinical features are summarized in Table S1a. The subtyping results were then integrated with clinical information to elucidate the relationships between molecular characteristics and therapeutic outcomes (Fig. 1a).

Overall, the proteomics analysis detected 8032 gene products as reliable identifications, which were used for further analysis (Fig. S1c, Table S2a, See STAR Methods). The number of proteins detected in each sample ranged from 1382 to 4268; more than 2500 proteins were detected in over 85% of samples (Fig. S1d), and 4766 proteins were detected in all samples (Fig. 1b). The number of proteins detected at the tumour sites was higher than that detected in the fallopian tubes. The relative abundance (iFOT) of the proteins after logarithmic transformation showed a distribution of approximately ten orders of magnitude (Fig. S1d). Principal component analysis (PCA) revealed well-separated distributions between fallopian tubes and tumour sites. In contrast, the three types of tumour sites exhibited no discernable differences, indicating similarity in tumour proteomes regardless of primary or metastatic sites (Fig. 1c).

To conduct proteomic subtyping, we first selected the top 2500 most abundant proteins detected in each sample, yielding a total of 6,424 proteins (Table S2B). We then selected proteins that were detected in more than ten percent of all samples (18/183 samples) with a coefficient of variation (CV) of abundance greater than 2, resulting in 1044 proteins (Fig. S1e, Table S2c). We scored the 1044 protein expression signatures and employed NMF consensus-clustering for subtyping (Fig. 1d, Fig. S1f, g, h). The NMF clustering yielded four subgroups with a maximum average silhouette of 0.78, namely, cluster 1 (C1, n = 40, 22%), cluster 2 (C2, n = 57, 31%), cluster 3 (C3, n = 48, 26%) and cluster 4 (C4, n = 38, 21%) (Fig. S1e, F). Gene Ontology (GO) [21] enrichment analyses further elucidated that C1 showed cell proliferation gene signatures (Fig. 1d, Table S2d). C2-enriched proteins were involved in immune response pathways; the C2 subtype also had elevated expression of chemotaxis proteins including ITGB2 and SERPINE1, which are required for immune cell migration [22–24]. C3-enriched proteins showed high association with cell-matrix adhesion and muscle system processes, such as LAMA5, TNXB and several collagen proteins. C4-enriched proteins were mainly involved in various metabolic pathways (Fig. 1d, Table S2d).

We assessed the correlation between proteomic subtypes of primary tumours and patient prognoses. Patients in C3 and C4 showed better prognoses, while those in C1 and C2 exhibited less favourable outcomes, as evidenced by the progression-free survival (PFS) and overall survival (OS) curves (Fig. 1e). Utilizing a similar subtyping procedure, the CPTAC and 2023 Ovarian Cancer (OC) validation cohorts can also be divided into 4 distinct proteomic subtypes with high average silhouette width (0.79, Fig. S1i, j, for CPTAC cohort; 0.78, Fig. S1l, m for 2023 OC cohort) and consistent molecular characteristics (Fig. 1f, Fig. S1k, Table S2e, f), further indicating the reliability of subtyping and the consistency in molecular characteristics. Intriguingly, the association of PFS with proteomic subtype was also observed in HGSC patients (Fig. S1n).

### The clinical relevance of the four proteomic subtypes

To assess whether the proteomic subtype could serve as an independent prognostic factor, we performed multivariate Cox regression analysis by controlling clinical characteristics. These included established factors such as age, residual tumour at surgery, timing of surgery, and FIGO stage, along with factors exhibiting prognostic correlation in univariate Cox analysis in our cohort (HE4, CA125). The results demonstrated that the proteomic subtype served as a robust and independent prognostic biomarker for evaluating OC patient OS (Table 1) and PFS (Table S1c).

**Table 1:** OS Multivariable Cox Regression Analysis (95% CI) for XY OC.

Characteristic	N	Event N	Univariate Cox		Multivariate Cox	
			HR (95% CI)^a^	*P*-value	HR (95% CI)^a^	*P*-value
**Age**	66			**0.051**		**0.008**
<=60		47	–		–	
>60		19	2.46 (0.99–6.077)		3.88 (1.09–13.88)	
**Residual tumour at surgery**	66					
R0		32	–		–	
R1		22	4.21 (1.30–13.71)	**0.017**	2.24 (0.49−10.29)	0.299
R2		12	4.17 (1.18–14.81)	**0.027**	0.83 (0.16−4.32)	0.826
**Timing of surgery**	66			0.269		0.945
PDS^b^		56	–		–	
IDS^c^		10	1.86 (0.61–5.62)		0.95 (0.22–4.06)	
**FIGO_stage**	66			**0.019**		0.905
I/II		22	–		–	
III/IV		44	11.0 (1.46–82.15)		0.85 (0.06–11.43)	
**CA125**	65			**0.018**		0.763
Low		37	–		–	
High		27	3.07 (1.21–7.81)		1.18 (0.39−3.60)	
**HE4**	53			**0.009**		0.186
Low		25	–		–	
High		27	4.36 (1.43–13.31)		2.48 (0.64−9.61)	
**Subtype**	66					
C3		22	–		–	
C1		14	5.75 (1.22–27.15)	**0.027**	6.23 (1.17–33.02)	**0.031**
C2		13	3.37 (0.61–18.41)	0.161	9.79 (1.47–65.20)	**0.018**
C4		17	1.88 (0.36–9.68)	0.451	1.79 (0.26–12.63)	0.555

^a^*HR* Hazard Ratio, *CI* Confidence Interval.

^b^*PDS* primary debulking surgery.

^c^*IDS* intervaldebulking surgery, refers to surgery after neoadjuvant chemotherapy (NACT).

# Events: 18; Global p−value (Log−Rank): 0.029064.

AIC: 132.01; Concordance Index: 0.79.

**Fig. 2:**
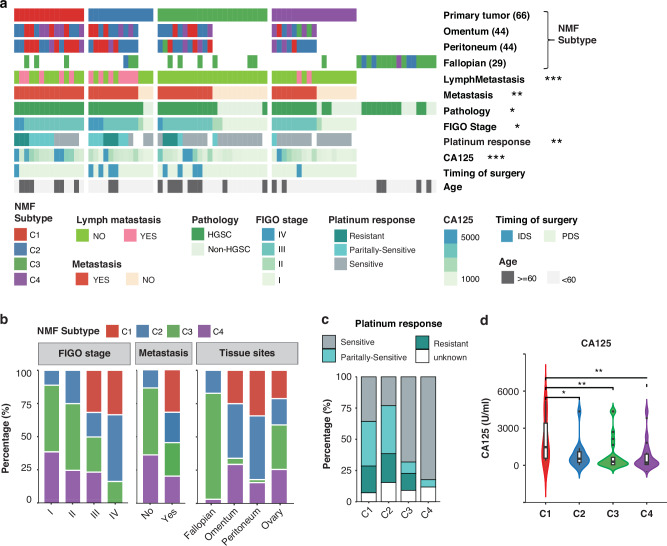
The four Proteomic subtypes show distinct clinical relevance. **a** The association of proteomic subtypes with 7 clinical variables. Kruskal-Wallis test was used for continuous variable CA125 (U/ml, the serum CA125 before debulking surgery or neoadjuvant chemotherapy). Fisher’s exact tests were used for other categorical variables (***p < 0.001, **p < 0.01, *p < 0.05). (Time of Surgery: PDS = primary debulking surgery, IDS = interval debulking surgery, refers to surgery after neoadjuvant chemotherapy (NACT)). **b** The distribution of samples with 4 Proteomic subtypes in different FIGO stages, metastasis, and tissue sites. **c** The distribution of samples with different platinum response in 4 Proteomic subtypes. **d** The distribution of serum CA125 level in 4 Proteomic subtypes. (Kruskal-Wallis test, **p < 0.01, *p < 0.05).

To further validate the clinical relevance, we analysed the correlations of clinical events with the four proteomic subtypes (Fig. 2a, b, Table S1a). Notably, among the four subtypes, patients with early-stage disease (FIGO stages I and II) and no distant metastasis predominantly clustered within the C3 subtype, suggesting a more favourable prognosis for this group (Fig. 2b). Intriguingly, 79.3% of fallopian tube samples were classified as C3 (Fig. 2a, b), suggesting a molecular relationship between the fallopian tube and C3 ovarian cancer.

Most patients with FIGO stage IV disease were concentrated within the C1 and C2 subtypes (83.3%, Fig. 2b). Patients experiencing relapse within 6 months, between 6 to 12 months, and beyond 12 months after completing prior platinum-based chemotherapy were categorized as platinum-resistant, partially platinum-sensitive, and platinum-sensitive, respectively (detailed definition see Additional Clinical Data). The C1 and C2 subtypes exhibited a higher proportion of patients with reduced chemotherapy response, including both platinum-resistant and partially platinum-sensitive cases (C1: 57.1% [8/14]; C2: 61.5% [8/13]; C3: 22.7% [5/22]; C4: 5.8% [1/17]) (Fig. 2c). Additionally, patients in the C1 subtype showed elevated serum CA125 levels (Fig. 2d). These correlations indicate a poorer prognosis for patients in the C1 and C2 subtypes. In contrast, the C4 subtype was associated with a favourable response to platinum-based therapy (Fig. 2c, 83% of C4 cases were platinum-sensitive) and had the second-highest proportion of early-stage patients (Fig. 2B), further supporting its link to the best prognosis. However, no strong relationship between the proteomic subtypes and other factors, including surgery, HE4 and ascites, was observed (Fig. S2a, b). Thus, we show that the four proteomic molecular subtypes exhibit distinct molecular features and clinical relevance.

### ScRNA-seq reveals the cell ecosystem of each proteomic subtype

**Fig. 3:**
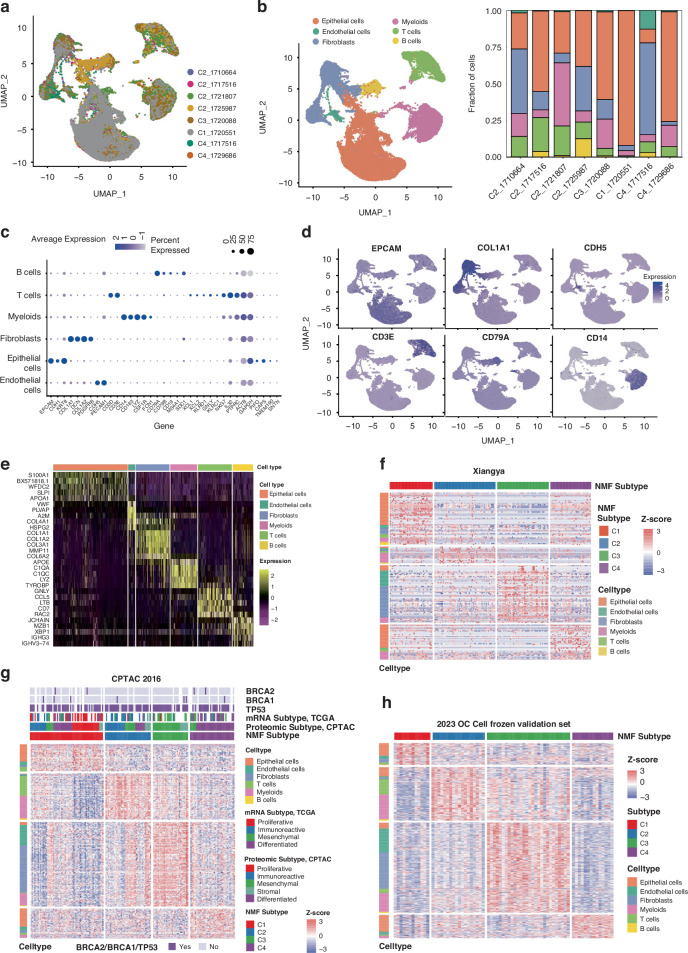
scRNA-seq analysis revealed the cell ecosystem of each proteomic subtype. **a** Uniform manifold approximation and projection (UMAP) showing the distribution of 8 scRNA-seq samples. **b** UMAP plot showing 6 cell clusters for 8 samples analysed by 10X scRNA-seq (Left). Ratio of 6 cell types relative to the total cell count per sample (Right, the total cell count was scaled to 1). **c** Bubble plot showing marker genes across 6 cell clusters. Dot size indicates fraction of expressing cells, coloured according to z-score scaled to expression levels. **d** UMAP plot showing expression levels of selected genes. **e** Heatmap showing the expression signature of top marker genes for 6 cell clusters. Expression signature of differential cell type specific proteins among 4 Proteomic subtypes from Xiangya OC proteomics (**f**), CPTAC 2016 OC cohort (**g**) and 2023 OC cohort (**h**). Colour of each cell indicates Z score (Log2 of global protein abundance scaled to proteomic expression standard deviations) of the protein in each sample. Annotations indicate 6 cell clusters (left) and Proteomic or mRNA subtypes or gene mutation status (up).

To better understand the molecular details of the four proteomic subtypes, we collected an additional 8 fresh primary tumours and performed scRNA-seq to study the cell ecosystem (Table S1b). We first performed proteomic profiling on the 8 samples (7 HGSCs and 1 clear cell carcinoma). NMF classified the 8 samples into C1 (1 sample, HGSCs), C2 (4 samples, 4 HGSCs), C3 (1 sample, 1 HGSC) and C4 (2 samples, 1 HGSC and 1 clear cell carcinoma) (Fig. 3a and Fig. S3a). High-quality transcriptomes of 40,710 single cells [25] were obtained based on the 10X Genomics platform (Table S3a). Using graph-based clustering, we first stratified them into 6 major cell populations: epithelial cells, endothelial cells, fibroblasts, T cells (including natural killer (NK) cells), B cells, and myeloid cells (including mast cells, macrophages, dendritic cells (DCs), and monocytes) (Fig. 3b). The composition and expression levels of key cell type markers confirmed the assignments for the cell clusters (*PECAM1* and *CDH5* for endothelial cells, *EPCAM, KRT8* and *CDH1* for epithelial cells, *DCN, COL1A1, COL1A2* and *PDGFRA* for fibroblasts, *CD3D* and *CD3E* for T cells, *CD79A, CD79B, CD19* and *MS4A1* for B cells and *LYZ, CD14, CD163* and *CSF1R* for myeloid cells) [26–28] (Fig. 3c, d). The labelling of the epithelial cell clusters was further supported by high copy number variation (CNV) scores (Fig. S3b).

The genes that were upregulated in each cell type (see STAR Methods) were considered as their distinctive signature genes (Fig. 3e, Table S3b). Subsequently, we assessed these cell type signature genes in the 183 original EOC samples, characterizing their cell types based on their protein expression levels. As shown in Fig. 3f and Table S4a, upregulated genes in C1 and C4 clusters exhibited similar cellular characteristics, predominantly enriched in epithelial-associated genes. C2 encompassed numerous immune cell-related genes, in consistency with the scRNA-seq analysis which revealed a high abundance of immune cells in C2 subtype samples (Fig. S3c). In contrast, C3 predominantly featured fibroblasts and endothelial genes (Fig. 3f). Similar gene signatures of cell clusters were also observed in the four subtypes in the CPTAC 2016 validation cohort and 2023 OC cohort (Fig. 3g, h, Table S4b). While we did not observe a clear correlation between BRCA mutations and the subtypes in CPTAC 2016 cohort. Based on cell type distribution and the molecular signatures (Fig. 1), we designated C1 (with the worst prognosis and highly expressed epithelial cell-related genes) as the malignant proliferative subtype, C2 (with the highest proportion of immune cells and highly expressed immune cell and cell-killing related genes) as immune infiltrating subtype, C3 (encompassing approximately 80% of the fallopian tubes and exhibiting high expression of fibroblast and endothelial signature genes) as the Fallopian-like subtype, and C4 (displaying a high percentage of epithelial-associated genes and enrichment in genes related to mitochondrial respiration) as the differentiated subtype.

### The immune infiltrating subtype is enriched with GZMK CD8+ T cells and MRC1 TAM-like macrophages

**Fig. 4:**
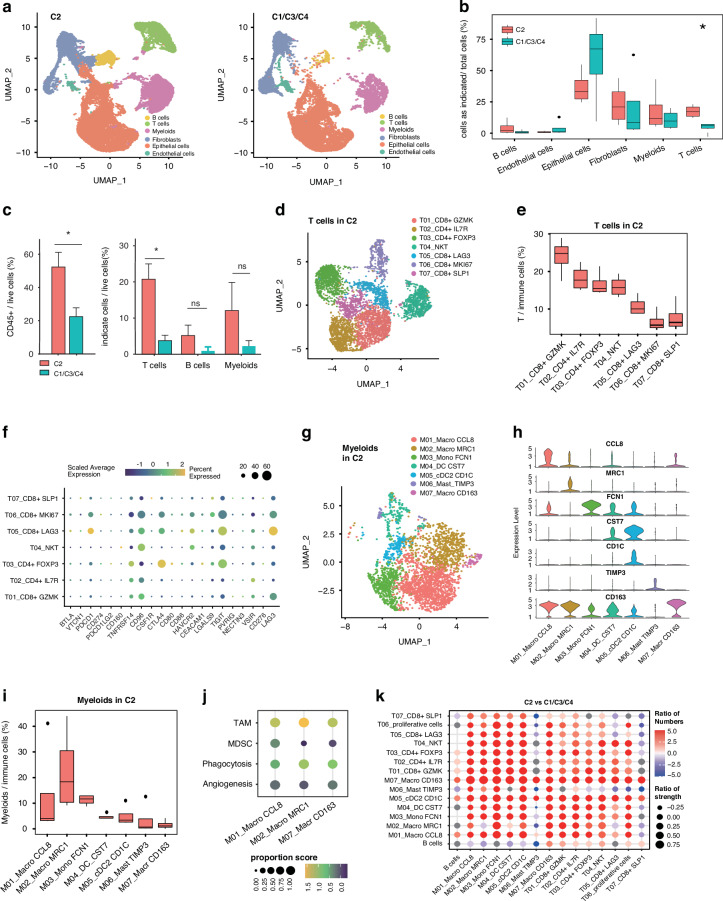
The immune infiltrating subtype (C2) is enriched with highly infiltrative GZMK CD8+ T cells and MRC1 TAM-like macrophages. **a** UMAP plot showing the distribution of 6 cell clusters in C2 (left, immune infiltrating subtype) and C1/C3/C4 proteomic subtypes (right). **b** Barplot showing percentage of 6 cell subpopulations in C2 and C1/C3/C4 subtypes. (*p < 0.05, error bar indicates ±SEM, two-sided Wilcoxon test) **c** Barplot showing the flow cytometry analysis of CD45^+^ cells (left), T cells (CD45^+^ CD3^+^), **b** cells (CD45^+^ CD19^+^) and Macrophage cells (CD45^+^ CD11b^+^) in C2 subtype compared with and C1/C3/C4 subtype. (*p < 0.05, error bar indicates ±SEM, two-sided Wilcoxon test) **d** UMAP plot showing 7 T cells subclusters from 4 C2 subtype patients. **e** Barplot showing percentage of 7 T cell subclusters in C2 subtype. **f** Bubble heatmap showing the expression of immune checkpoint genes across 7 T cell subclusters. **G** UMAP plot showing myeloid cells from 4 C2 patients. **h** Violin plot showing the expression of selected marker genes across 7 myeloid cell clusters. **i** Barplot showing the percentage of myeloid cells subclusters in C2 subtype. **J** Bubble heatmap showing gene signature score of TAMs, MDSCs, Angiogenesis and Phagocytosis in selected macrophage subclusters as indicated. Bubble size represents the proportion of cells with enrichment gene score > 1. The colour of the circle represents the enrichment gene scores. **k** The ratio of interaction strength and numbers of immune cells as indicated in the immune infiltrating subtype compared to those of non-immune subtypes. Bubble size represents the ratio of interaction strength. The colour of the circle represents the ratio of interaction numbers.

Consistent with the gene and protein signatures, the immune infiltrating C2 subtype contained a high percentage of immune cell infiltration (Fig. S4a), while the clinical outcome is worse in our proteomic analyses. We next stratified the 8 scRNA-seq samples into 4 immune (C2) and 4 non-immune groups (C1, C3 and C4) according to our proteomic subtypes, and analysed the immune cell subpopulations. The immune infiltrating subtype contained higher percentages of CD45+ immune cells (Fig. S4b–d), T cells and CD14+ myeloid cells (Fig. 4a, b). Fluorescence-activated cell sorting (FACS) analysis further verified the immune cell percentages. As shown in Fig. 4c, CD45+ cells, particularly T cells, were indeed enriched in immune infiltrating subtype tumours, consistent with the scRNA-seq results. B-cell and myeloid cell populations showed small and statistically insignificantly higher percentages in C2 tumours (Fig. 4c, Fig. S4e).

To gain a deeper understanding of immune infiltration in OC, we analysed 4667 T cells from the four C2 samples and classified them into 7 subclusters (Fig. 4d). We identified two CD4+ subclusters (T02 and T03) with expression of *IL7R* and *FOXP3* and four CD8 + T-cell subclusters (T01, T05, T06 and T07) with expression of *GZMK, LAG3, MKI67* and *SLP1* (Fig. 4d, Fig. S4f, g, Table S5a). We also identified an NKT cluster in the T-cell population that was marked by the expression of cytotoxic genes *NKG7, GNLY* and *GZMB* [5, 29] (Fig. 4d, Fig. S4f, g, Table S5a), consistent with the notion that NK cells transcriptionally resemble T cells. Further interrogation of the T-cell subpopulations in the immune infiltrating subtype revealed that the CD8 + GZMK T01 subcluster and the CD4 + IL7R T02 subcluster showed significantly higher percentages in the immune infiltrating subtype than in the other Proteomic subtypes (Fig. 4e, Fig. S4h). The CD8 + GZMK T01 subcluster has been characterized in several studies and is presumed to be effector memory T (Tem) cells based on its coexpression of granzymes, including *GZMA, GMZH*, and cytokine *CCL5* [5, 30, 31]. The CD4 + IL7R T02 subcluster has been suggested to be naïve T cells with high coexpression of *CCR7* [32, 33].

The effects of T cells are maintained by homoeostatic regulation of activation and exhaustion. We thus evaluated the expression of immune inhibitory checkpoint genes that determine the cytotoxic function of T cells. We found that the CD8 + LAG3 T05 subcluster, which accounted for approximately 9% of immune cells, expressed the highest levels of the *PDCD1* and *LAG3* genes (Fig. 4f). In addition, *CTLA4* was highly expressed in the CD4 + FOXP3 T03 subcluster, which only accounted for 16% of cells in tumours. In contrast, CD8 + GZMK T01, the most abundant subcluster, expressed low levels of *PDCD1* (PD1), *CD274* (PDL1), *PDCD1LG2* (PDL2) and *CTLA4* but a moderate level of LAG3 (Fig. 4f).

Myeloid cells, which include DCs, macrophages, mast cells, and monocytes, were the second most enriched immune cells in the immune infiltrating subtype. We next employed a similar algorithm to interrogate the myeloid subpopulation and obtained 7 subclusters [5] (Fig. 4g, Fig. S4i). DCs and macrophages share overlapping molecular profiles [34, 35]. Subclusters 4 (M04) and 5 (M05) were defined as conventional DCs (cDCs) characterized by high expression of *CST7, HMGA, CD1C* and *CLEC10A* [29]. M03 was defined as monocytes based on the expression of the key markers *FCN1, S100A* and *VCAN* [5, 29]. M06 was defined as the mast cell subcluster based on the expression of the genes *CSF1* and *TIMP3* [29]. The remaining clusters (M01, M02 and M07) were identified as macrophages based on their high expression of the characteristic macrophage genes *CD68, CD163, CCL8* and *MRC1* (Fig. 4h, Fig. S4i, Table S5b). Among subclusters, the macrophage CCL8 M01 and MRC1 M02 subclusters showed higher proportions in all myeloid cells (Fig. 4i, Fig. S4j). The MRC1 M02 subcluster, which contained high expression levels of *HLA-DRs* (members of the MHC II family), has been suggested to have enhanced interactions with CD4 + T cells [36].

We then determined differentially expressed pathways in the myeloid compartment between C2 and C1/C3/C4. In the C2, myeloid overexpressed genes were enriched in myeloid leucocyte activation, especially T cell activation. Gene products such as *C1QA, CCL5*, and antigen processing via MHC class I (*HLA-A, HLA-B*) were mainly recognized by CD8+ T cells. In contrast, MHC class II gene products, (*HLA-DMA, HLA-DMB, HLA-DOA, HLA-DPA1, HLA-DPB1, HLA-DQA2, HLA-DQB1, HLA-DRA, HLA-DRB5, HLA-DRB6*) which are mainly recognized by CD4+ T cells, were enriched in C1/C3/C4 (Fig. S4l and Table S5d).

Tumour-associated macrophages (TAMs) and myeloid-derived suppressor cells (MDSCs) are distinct types of myeloid [29, 37]. Increasing evidence indicates that MDSCs are linked to tumour progression and poor prognosis [37–39]. We initially analysed TAM and MDSC scores across several major myeloid populations in C2 patients using scRNA-seq data (Table S5c). Our findings revealed that M03, characterized by a high MDSC score, was associated with pro-tumour property (Fig. 4j, Figure Sk). The functional status of TAMs was further evaluated based on M1/M2 polarization, which has opposing activities. While, M1 and M2 gene signatures [40] (Table S5c) can coexist within TAMs [41] which was also observed in our data (Fig. S4k). Thus, relying solely on the M1/M2 signature score may lead to inaccurate functional predictions. We then evaluated the myeloid immune response of C2 using angiogenic and phagocytic signatures (Table S5C), which were previously established to identify functional macrophages in colorectal cancer, liver cancer and pan-cancer single-cell analyses (Table S5c) [29, 41]. The phagocytosis-like macrophages (M01, M02 and M07) possessed high phagocytosis-related gene scores and were associated with anti-tumour phenotype (Fig. 4j, Fig. S4k), implying a potentially favourable antitumor immune response of myeloid cells in the C2 subtype. Immune cell-cell interaction analysis showed enhanced interactions between myeloids and T cells for patients in the immune infiltrating subtype but not in non-immune subtype patients (Fig. 4k, Fig. S4m). Notably, M01, M02 and M07 exhibited significantly increased interactions with CD8 + GZMK T01 cells in the immune infiltrating subtype compared with non-immune subtypes (Fig. 4k, Fig. S4n).

### Identification of CD40 as a potential therapeutic target for the C2 immune infiltrating subtype

**Fig. 5:**
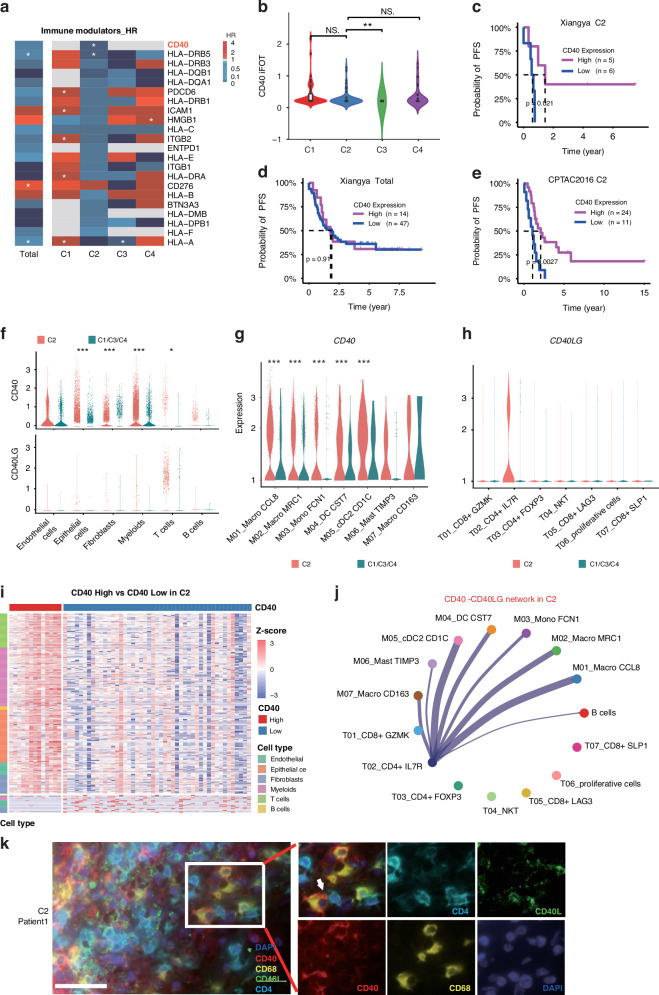
CD40 is a potential therapeutic target for the immune infiltrating subtype. **a** The heatmap showing hazard ratio (HR) values of the 22 immune modulator genes calculated using elastic-net cox proportional-hazards (CoxPH) model in each subtype. Red represents higher survival risk and blue represents lower survival risk. (* p < 0.05). **b** CD40 protein abundance in 4 Proteomic subtypes. (Kruskal-Wallis test). **c** The association of CD40 protein expression with PFS in NMF C2 immune infiltrating subtype of Xiangya OC proteomics. (Log-rank test) **d** The association of CD40 protein expression with PFS in all patients of Xiangya OC proteomics. **e** The association of CD40 protein expression with PFS in immune infiltrating subtype C2 of CPTAC OC proteomic validation cohort. **f** Violin plot showing the expression of *CD40* and *CD40LG* across 6 cell subpopulations in immune infiltrating and non-immune subtypes. **g** Violin plot showing the expression of *CD40* across 7 Myeloid subpopulations in immune infiltrating and non-immune subtypes(*p < 0.05, ***p < 0.001, Wilcoxon test). **h** Violin plot showing the expression of *CD40LG* across 7 T cell subpopulations in immune infiltrating and non-immune subtypes. **i** Expression signature of differential proteins between CD40 high and low expressed patients in C2 from Xiangya OC proteomics. Colour of each cell indicates Z score (log2 of global protein abundance scaled to proteomic expression standard deviations) of the protein in each sample. Annotation indicates CD40 expression level (up) and marker genes of 6 cell clusters (left). **j** The inferred CD40-CD40LG networks of immune cells calculated by cellchat. **k** Representative multispectral immunofluorescent images of CD40, CD40L, CD4, CD8, CD68 and DAPI in patients’ tumour sample of C2. Scale bar, 50 μm.

Despite remarkable progress in immunotherapy for various cancers [42, 43], the outcomes in EOC have been poor. The less-than-satisfactory results from clinical trials involving immune checkpoint inhibitors, such as anti-PD-L1 avelumab (NCT02718417, NCT02580058), atezolizumab (ENGOT-ov29-GCIG(ATALANTE), NRG-GY009) and anti-CTLA4 ipilimumab (NRG-GY003), prompted the exploration of alternative immunotherapeutic target in EOC [44]. To find potential immunotherapeutic targets, we conducted a comprehensive evaluation prognostic significance of immune modulator genes using the elastic-net Cox proportional hazards (CoxPH) model for each subtype [45, 46]. Our analysis identified 2 genes that exhibited a significant association with improved PFS specifically within the immune infiltrating subtype (p-value of C2 < 0.05), while showing a less pronounced impact in other subtypes (p-value > 0.1) (Fig. 5a and Table S6a). Notably, the high expression of the myeloid marker CD40 emerged as a particularly significant gene linked to enhanced survival specifically within the immune subtype.

CD40 expression exhibited no significant difference among C1, C2 and C4 (Fig. 5b), but was rarely detected in C3. Univariate survival analysis showed that high expression of CD40 was associated with better PFS for the C2 subtype (log-rank test, P = 0.009) (Fig. 5c), but showed no prognostic relevance with the non-C2 subtype and total patient population (log-rank test, *P* = 0.95) (Fig. 5d, Fig. S5a). Similar correlations were also validated in the CPTAC dataset. A positive correlation with prognosis was found only in the C2 immune infiltrating subtype patients (Fig. 5e, Fig. S5b, c).

CD40, a receptor from the tumour necrosis factor receptor (TNFR) superfamily, binds with its ligand, CD40L. This interaction triggers a cascade of signalling pathways that promote immune cell activation, proliferation, differentiation, and survival [47]. The scRNA-seq analysis showed that *CD40* was highly expressed in myeloid cells in the immune infiltrating subtype, whereas *CD40LG*, encoding the CD40 ligand (CD40L), was only expressed in T cells (Fig. 5f). Additionally, *CD40* showed higher expression in the M01-M05 myeloid subclusters (Fig. 5g), whereas *CD40LG* elevated specifically in the T2_CD4 + IL7R subcluster within the immune-infiltrating subtype (Fig. 5h). To further investigate the proteomic signatures of patients with differential CD40 expression in the C2 cluster, we assigned the patients into CD40 high and low groups and compared the composition of immune cell types in which differential genes were expressed. Consistently, the CD40 high-expressing subgroup had increased levels of T-cell and myeloid cell gene signatures than the CD40 low-expressing subgroup (Fig. 5i) which may be a mechanism underlying the better prognosis.

Since CD40 expression was correlated with better survival in the immune infiltrating subtype, we carried out cell-cell interaction network analysis [48] based on the CD40-CD40LG. As shown in Fig. 5j, myeloid subclusters (M01, M02, M05) exhibited extensive interactions with CD4 + IL7R T cells (T02). In contrast, this interaction was not detected in the non-immune subtypes due to the low expression of CD40L. To further explore the role of CD40-CD40L in different scenarios, we performed multiplexed immunofluorescence analyses on the tissue section. Our result revealed that CD40 + CD68+ macrophages were closely associated with CD40L + CD4+ T cells specifically in C2 subtype patients, a pattern not commonly observed in other subtypes (Fig. 5k, Fig. S5d). Together, our results suggest that the CD40-CD40L interaction may occur between myeloid cells and CD4+ T cells, and the lack of CD40L expression in CD4+ T cells in the C1/C3/C4 subtypes makes it unable to activate the CD40 pathway.

### The TYMP inhibitor, TAS102, could be a new selective chemotherapy for non-C2 EOC

**Fig. 6:**
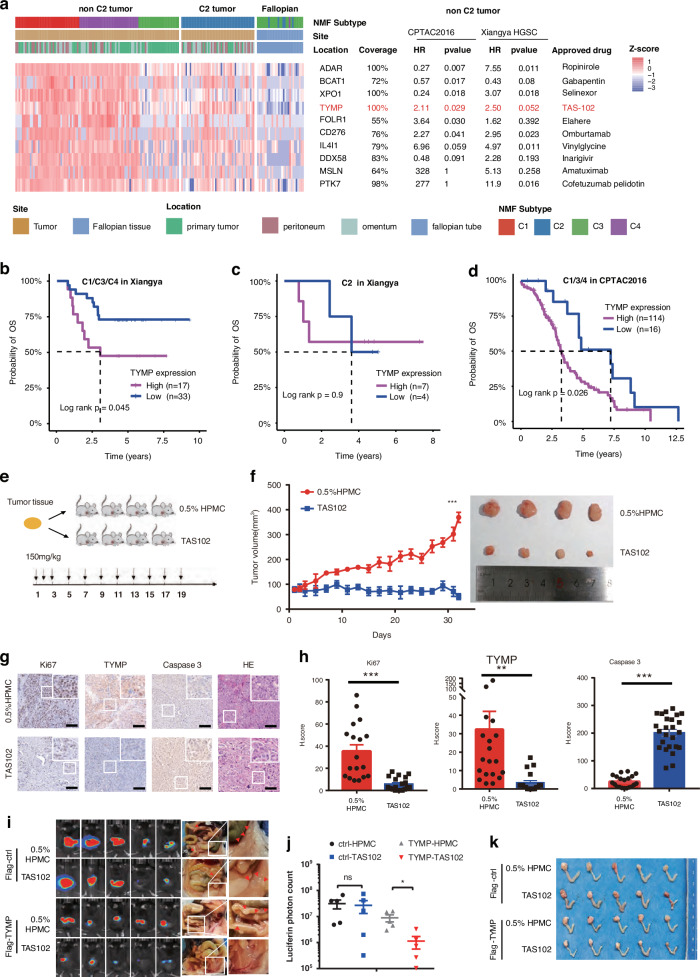
Druggable candidates, particularly TAS102, could be a new selective chemotherapy for ovarian cancer. **a** The heatmap of expression signature of selected druggable candidates between tumours and fallopian tissues. Annotation indicates the coverage (detected frequency) and cox analysis in non-C2 HGSC tumours and approved drugs for each gene. The association of TYMP protein expression with OS in non-C2 (C1/C3/C4) patients of Xiangya OC proteomics (**b**) and CPTAC2016 OC proteomics (**c**). Log-rank test. **d** The association of TYMP protein expression with OS in C2 patients of Xiangya OC proteomics. Log-rank test. **e** Schematic diagram of NOD-SCID mice implant PDX model. Time schedule and concentration (150 mg per kg, oral) of treated TAS102 as indicated. **f** Growth curves of PDX tumours treated with 0.5% HPMC or TAS102. The mice with tumours were randomly divided into two groups treated with 0.5% HPMC or TAS102 after the tumour volumes reaching to 60–100 mm^3^. Tumour volumes were measured at indicated time points. (n = 4, error bars, SEM; ***P < 0.001, two-sided, unpaired t test). Tumours were isolated from individual mice at the end of the treatments (right). **g** Representative images of IHC staining with cell proliferation marker Ki67, TYMP, apoptosis marker caspase 3, and HE in tumour samples derived from PDX in NOD-SCID mice. Scale bar, 100 μm. **h** Quantitation of the histologic score (H score) of indicated markers from three random areas of four individual mice in different groups as indicated (***P < 0.001, **P < 0.01, two-sided, unpaired t test). **i** C57BL/6j mice were orthotopically inoculated with ID8^Luc+^ cells with or without TYMP expression and subsequently treated with HPMC or TAS102 (150 mg per kg, oral). Representative luminescent images and abdominal metastases (right) of mice bearing vector or TYMP overexpressing ID8^Luc^ tumour treated with HPMC (Control) or TAS102 at day 21 are shown.j The luciferin photon count of mice treated with HPMC or TAS102 at day 21. (*P < 0.05, two-sided, unpaired t-test). **k** Representative images of orthotopic tumours isolated from individual mice at the end of the treatments.

Platinum-based treatment remains the primary chemotherapy for EOC patients. The limited therapy options and high percentage of recurrence contribute to the overall poor prognosis. To further interrogate new therapeutic targets, we mapped druggable targets for non-immune patients (C1/C3/C4) who are ineligible for immunotherapy. We selected proteins derived from an in-house database encompassing various cancers. We screened for proteins that were highly expressed in epithelial ovarian cancer (EOC) tumour tissues compared to adjacent normal tissues. Subsequently, we identified proteins targeted by drugs approved for clinical application or under clinical trials as our candidates (Fig. 6a).

TYMP, which was found to be highly expressed in tumour tissues, especially in non-immune subtypes (Fig. S6a), and exhibited a significant negative association with the prognosis in HGSC patients of non-immune subtypes in both Xiangya and CPTAC 2016 cohort (Fig. 6a, b, c, S6b). No strong relationship between TYMP and prognosis in the immune subtype was found (Fig. 6d, S6c, d). The TYMP-targeting drug TAS-102, a trifluridine-tipiracil hydrochloride mixture, is currently approved for treating advanced colorectal cancer and gastric cancer [49, 50] after at least two lines of chemotherapy [51–54]. To evaluate the drug efficacy in non-immune subtype tumours, we selected a non-immune tumour with high TYMP expression (tumour#Exp031854) (Fig. S6e) and established a patient-derived xenograft (PDX) model. TAS-102 administration to the PDX model resulted in a significant reduction in tumour size compared to vehicle treatment, indicating a potent antitumor effect of TAS-102 (Fig. 6e, f, Fig. S6f). Furthermore, histological immunochemistry revealed strong staining of TYMP and the proliferation marker Ki67 in untreated tumour samples. In TAS102-treated tumours, TYMP and Ki67 staining decreased, and caspase 3 increased, demonstrating that TAS102 effectively inhibited tumour growth and induced apoptosis (Fig. 6g, h). A slight reduction in body weight in the TYMP-treated groups was observed, indicating a possible side effect of TAS102 that was observed similar to most chemotherapeutic drugs (Fig. S6g).

To further explore the role of TYMP, we analysed its gene expression from scRNA-seq data and found that TYMP was differentially expressed in epithelial cells, which are much more prevalent in tumours with non-immune subtypes compared to immune subtypes. Notably, TYMP expression was significantly higher in malignant epithelial cells (Fig. S6h), suggesting a critical role for TYMP in tumour cells. Consequently, we generated TYMP-overexpressing ID8 (mouse) ovarian cancer cell lines and established an orthotopic syngeneic mouse model [55] by inoculating ID8^Luc+^ cells into the ovarian bursal cavity of C57/BL6 mice (Fig. S6i). TAS102 treatment effectively reduced tumour burden and metastasis, particularly in TYMP-overexpressing ID8^Luc^ mice, as evidenced by lower luciferin intensity and Ki67 staining (Fig. 6i–k, Fig. S6j). However, slight weight loss was observed in both vector and TYMP mice following prolonged and intensive TAS102 treatment (Fig. S6k), indicating that TAS102, like other chemotherapeutic agents, can manifest toxicity.

## Discussion

Employing unbiased proteomics, we examined representative EOC samples, establishing a proteomic atlas that delineates four distinct molecular subtypes. By integrating proteomic and scRNA-seq analyses, we validated the cellular landscape and molecular characteristics of each proteomic subtype, establishing a connection between proteomics-based features and cell-cell mediated microenvironments. An in-depth function-driven analysis unveiled CD40 as a subtype-specific (the immune infiltrating type) favourable prognostic factor. Further investigation suggested that CD40-based immunotherapy held promise for this subtype, indicating a potential avenue for precision and personalized therapy in EOC.

The four subtypes we identified demonstrate partial consistency with the existing proteomics classification for HGSC, while less than ~50% consistency with transcriptomic subtypes [3, 56–58]. Moreover, we noticed that patients with early-stage ovarian cancer, regardless of histological type, clustered into the same subtype, indicating a common molecular characteristic across different histological types. This also highlights distinct molecular differences between early-stage and advanced-stage ovarian cancer. Additionally, paired primary and metastatic tumour samples from the same patient were assigned to different proteomic subtypes, reflecting the molecular heterogeneity of EOC and emphasizing the need for combination therapeutic strategies. The origin and pathogenesis of EOC remain controversial, with two main hypotheses based on morphological, pathological [59, 60], and epidemiological [61] evidence. The first hypothesis suggests that OC originates from the ovarian surface epithelium, while the second proposes its emergence from the fallopian tubes. Our proteomic analysis shows that 79% of normal fallopian tube tissues were classified into the C3 subtype, suggesting that a subset of patients, specifically those with the C3 subtype, may exhibit a fallopian tube-like phenotype. However, further investigation, including cell-based lineage tracing, is needed to address questions about the exact cell origin.

The C2 subtype, identified as the immune infiltrating subtype, presents notable features. This subtype shows consistency with previous findings that observed a significant presence of tumour-infiltrating lymphocytes (TILs) in HGSC [3, 62] (Fig. 4). The immune microenvironment, including factors like M1/M2 macrophage polarization, can influence overall survival in ovarian cancer [63]. Despite high levels of T cell and macrophage infiltration observed in the C2 immune-enriched subtype, this group has a poor prognosis (Fig. 1). Effective T cell activation requires not only antigen recognition and T-cell receptor (TCR) engagement but also strong co-stimulatory signals and cytokine secretion, which are critical for T cell proliferation and function. Our findings on the association between CD40 and prognosis suggest that reduced CD40-CD40L expression may contribute to immune microenvironment inactivation and the poor outcomes seen in the immune subtype. However, definitive evidence of fully functional T cells in this subtype is still under investigation. Additionally, the C2 subtype includes a higher proportion of patients who are resistant or only partially responsive to platinum-based chemotherapy (Fig. 2c, Fig. S2c), indicating a potential intrinsic interaction between the immune microenvironment and drug sensitivity. Further, cell-based assays and mechanistic studies are needed to explore these interactions in greater detail.

Patients with EOC have shown limited benefits from PD-1 blockade immunotherapy [64]. The lack of response to PD-1 blockade is attributed to factors such as the unique composition of tumour-infiltrating immune cells, the heterogeneous immune microenvironment, and the low abundance of PD-1 [65–67]. Although previous investigations have identified expression of PD-1 and CTLA-4 in EOC tumours by immunochemistry staining [68, 69], our proteomics analysis did not detect PD-1 and CTLA-4 expression (Table S4a), indicating their potentially extremely low levels of expression in these samples. Furthermore, our scRNA-seq analysis revealed that immune infiltrating subtype tumours had a high percentage of infiltrating GZMK CD8 T cells which exhibited very low levels of PD-1, CTLA-4, and other immune checkpoint genes. This finding presents a significant challenge to the effectiveness of PD-1- and CTLA-4-based immunotherapies in treating EOC.

The discovery of CD40 as a specific prognostic factor for the immune infiltrating subtype illuminates new avenues for immunotherapy in EOC. CD40, part of the TNF receptor superfamily, is found on various immune cells, including dendritic cells (DCs) and macrophages. It interacts with its ligand, CD40L, present on activated T cells [68, 70, 71]. Our scRNA-seq analysis further revealed that CD40 is expressed in various macrophage subtypes, while CD40L is predominantly present in IL17R CD4 + T2 cells (Fig. 5). The significant CD40-expressing macrophage subclusters M01 and M02 and CD40L-expressing IL17R CD4 + T2 cells suggest that the macrophage-T-cell regulatory network plays a pivotal role in shaping the immune microenvironment. In addition, the higher phagocytosis scores of M01 and M02 (Fig. 4J) further amplify the antitumor effects of CD40 within the immune-infiltrating subtype. A previous study has indicated that both CD28 and CD40 are crucial for T-cell stimulation and can synergistically enhance PD-1 blockade efficacy [68]. However, in our analysis, CD28 protein expression was not detected in the immune infiltrating subtype, possibly due to the sensitivity limits of mass spectrometry-based detection methods. Given these findings, we propose that CD40 agonists might offer therapeutic benefits to patients with the immune infiltrating subtype of OC. While CD40 agonists have historically been associated with considerable toxicity, the development of tumour-targeted immunomodulators such as bispecific antibodies may offer a more tolerable means of targeting CD40 [72]. Nonetheless, the actual clinical efficacy and means of targeting CD40 for the immune subtype remains to be confirmed through further validation in tumour models.

Platinum-based drugs exert their therapeutic effects by inducing DNA breaks through crosslinking with DNA strands, leading to the disruption of both DNA replication and transcription processes. On the other hand, 5-Fluorouracil (5-FU), a pyrimidine analogue, achieves its effects by inhibiting thymidylate synthase, a crucial enzyme in DNA synthesis. While 5-FU is a widely used chemotherapy agent for colorectal and breast cancers, its efficacy in treating ovarian cancer, particularly in High-Grade Serous Carcinoma (HGSC), is notably less pronounced. TYMP plays a pivotal role in nucleotide metabolism. High levels of TYMP expression can diminish the effectiveness of 5-FU, resulting in reduced efficacy against cancer. Furthermore, we observed the high expression of TYMP linked to a poorer prognosis in non-immune EOC, particularly in HGSC (Fig. 6, and Fig. S6,). This suggests that TAS102, which targets TYMP combined with 5-Fu, could be considered a potential therapeutic strategy in HGSC.

Overall, our work integrates proteomics-based and scRNA-seq to reveal molecular subtypes and their associations with clinical outcomes for EOC. Furthermore, we propose the use of CD40-based immunotherapy for patients with immune infiltrating EOC patients and identify additional potential therapeutic targets for non-immune EOC patients. Our research offers a valuable resource for further exploration into the molecular subtype-specific microenvironments of EOC. This, in turn, could facilitate the development and application of personalized and precision therapies in the treatment of EOCs.

## Methods

### Sample collection

The specimen used in this study were collected from ovarian cancer patients who underwent laparoscopy or primary debulking surgeries between 2013 and 2019 from Xiangya Hospital of Central South University in China. All samples were approved by the Medical Ethics Committee of Xiangya Hospital, Central South University. Written informed consent was obtained from all participants.

We collected specimens before any treatment with written informed consent provided by all participants. Each tumour specimen was approximately 1 cm^3^ in size and weighed between 100 mg and 200 mg, in general. The specimens were frozen in liquid nitrogen within 30 min after resection and stored at −80 °C until used for further analysis. The remaining tumour tissues were formalin-fixed, paraffin-embedded (FFPE), stained and reviewed by pathologists to confirm the histological type (detailed information in supplementary information).

### Mass spectrometry analysis

Digested peptides redissolved in 0.1% formic acid were injected into an in-house packed reversed-phase C18 precolumn (2 cm × 100 μm; particle size, 3 μm; pore size, 120 Å) and then eluted using a linear gradient of 6–40% Mobile Phase B (acetonitrile and 0.1% formic acid) for 150 min. The resulting peptides were analysed on a Fusion mass spectrometer (Thermo Fisher Scientific) coupled with an Easy-nLC 1000 HPLC nanoflow system (Thermo Fisher Scientific). To acquire mass spectra, data-dependent mode was applied by carrying out a Full MS scan (AGC target 3 × 10^6^ ions, maximum injection time 20 ms, 300–1400 m/z, R = 60,000 at 200 m/z) followed by up to 20 tandem MS/MS scans with high-energy collision dissociation (target 2 × 10^3^ ions, max injection time 40 ms, isolation window 1.6 m/z, normalized collision energy of 27%), detected in the Iontrap (R = 15,000 at 200 m/z). Dynamic exclusion time was set to 18 s. All data was acquired using the Xcalibur software (Thermo Fisher Scientific).

### Chromium 10x Genomics library and sequencing

Single-cell suspensions were loaded to 10x Chromium to capture 5000 single cell according to the manufacturer’s instructions of 10x Genomics Chromium Single-Cell 5’VDJ kit (V5.2). The following cDNA amplification and library construction steps were performed according to the standard protocol. Libraries were sequenced on an Illumina NovaSeq 6000 sequencing system (paired-end multiplexing run, 150 bp) by LC-Bio Technology co.ltd., (HangZhou, China) at a minimum depth of 20,000 reads per cell.

### Cell culture and cytotoxicity assays

ID8 cells were cultured in DMEM medium (Gibco, #8120506) and supplemented with 10% FBS (Biological Industries, #18224477). Cell lines were maintained at 37 °C in an atmosphere containing 5% CO_2_. For cell cytotoxicity assay, 3000 cells were plated into a 96-well tissue culture plate the day before and then treated with the indicated compounds for another 48 h, and then incubated with Cell Counting Kit-8 (CCK-8) (Bimake, #B34304) agents for 1 hr. The 450 nm absorbances were then measured and calculated for the viability curve by normalizing all the values to the control group.

### Multispectral immunofluorescence (mIF) microscopy

Multispectral immunofluorescence (mIF) microscopy was performed on 4 μm formalin-fixed paraffin-embedded (FFPE) sections from patient ovarian tumours. Slides were heated at 63 °C for 1 hour and deparaffinized by immersing them in xylene, three washes 5 min each and rehydrated by immersing them in ethanol grades step. The manual mIHC staining kit (PerkinElmer, USA) was used. The staining procedure consists of consecutive rounds of antigen retrieval, blocking, staining with primary antibody, incubation with secondary HRP-labelled antibody, Opal dye development and antibodies denaturation. Finally, the sections were counterstained with DAPI and mounted. The multiplexed panels CD40/CD40L/CD4/CD8/CD68 was validated and used. The following primary Antibody were used: CD40 (1:500, ab224639, Abcam), CD40L (1:500, 66502-1-Ig, Proteintech), CD4 (Abcarta), CD8 (Abcarta), CD68 (Abcarta). Multiplex stained slides were imaged using the TissueFAXS system (TissueGnostics Asia Pacific Limited, Austria).

### Statistical analysis for proteomics

NMF consensus clustering method implemented in the NMF R-package was applied for subtyping in all datasets. The progression-free survival (PFS) and overall survival (OS) were defined as the duration from the last chemotherapy to the disease progression or death from cancer respectively. Kaplan–Meier survival analysis and the log-rank p-value was calculated using the Survminer R package (version 0.4.9). Univariate Cox proportional hazards regression analysis (including confidence intervals and p-values) was conducted by fitting all possible prognostic factors using the “survival::coxph” function. Multivariate Cox model that included age, residual tumour at surgery, timing of surgery, FIGO stage (those four were established as reliable prognostic factors before), and CA125 and HE4 (those two exhibited the prognostic correlation in univariate cox analysis) and the subtypes as covariates. Sample statistics used for PFS, OS and other clinical relevance were patients with primary tumour sites. All P-values less than 0.05 were considered to indicate statistical significance. Gene Ontology (GO) analysis between Proteomic subtypes was performed by the clusterProfiler package (version 4.2.2).

## Supplementary information

supplementary information

Figure S1

Figure S2

Figure S3

Figure S4

Figure S5

Figure S6

Table S1

Table S2

Table S3

Table S4

Table S5

Table S6

A reproducibility checklist

## Data Availability

Proteomics data generated in this study will be available in Table S2 integrated with demographic and clinical data in Table S1A, B. Source proteomic data of CPTAC OC proteomics in this study can be accessed through the CPTAC data portal or from the matrix Table S2 [3]. RNAseq data of GDC TCGA Ovarian Cancer in this study can be accessed through xenabrowser (https://gdc-hub.s3.us-east-1.amazonaws.com/download/TCGA-OV.htseq_fpkm.tsv.gz; Full metadata). The rest of the data available in the article can be found in the supplemental information or from the authors upon request.

## References

[1] SEER cancer statistics. Surveillance Research Program, National Cancer Institute.: SEER cancer statistics. Surveillance Research Program, National Cancer Institute.; 2023. Available from: https://seer.cancer.gov/explorer/.

[2] Cancer Genome Atlas Research N. Integrated genomic analyses of ovarian carcinoma. Nature. 2011;474:609–15. 21720365 10.1038/nature10166 PMC3163504

[3] Zhang H, Liu T, Zhang Z, Payne SH, Zhang B, McDermott JE, et al. Integrated proteogenomic characterization of human high-grade serous ovarian cancer. Cell. 2016;166:755–65. 27372738 10.1016/j.cell.2016.05.069 PMC4967013

[4] Izar B, Tirosh I, Stover EH, Wakiro I, Cuoco MS, Alter I, et al. A single-cell landscape of high-grade serous ovarian cancer. Nat Med. 2020;26:1271–9. 32572264 10.1038/s41591-020-0926-0 PMC7723336

[5] Hornburg M, Desbois M, Lu S, Guan Y, Lo AA, Kaufman S, et al. Single-cell dissection of cellular components and interactions shaping the tumor immune phenotypes in ovarian cancer. Cancer Cell. 2021;39:928–44.e6. 33961783 10.1016/j.ccell.2021.04.004

[6] Anadon CM, Yu X, Hänggi K, Biswas S, Chaurio RA, Martin A, et al. Ovarian cancer immunogenicity is governed by a narrow subset of progenitor tissue-resident memory T cells. Cancer Cell. 2022;40:545–57.e13. 35427494 10.1016/j.ccell.2022.03.008 PMC9096229

[7] Brachova P, Mueting SR, Carlson MJ, Goodheart MJ, Button AM, Mott SL, et al. oncomorphic mutations predict resistance to platinum- and taxane-based standard chemotherapy in patients diagnosed with advanced serous ovarian carcinoma. Int J Oncol. 2015;46:607–18. 25385265 10.3892/ijo.2014.2747 PMC4277253

[8] Kang HJ, Chun SM, Kim KR, Sohn I, Sung CO. Clinical relevance of gain-of-function mutations of p53 in high-grade serous ovarian carcinoma. PLoS One. 2013;8:e72609. 23967324 10.1371/journal.pone.0072609 PMC3742716

[9] Kigawa J, Sato S, Shimada M, Takahashi M, Itamochi H, Kanamori Y, et al. p53 gene status and chemosensitivity in ovarian cancer. Hum Cell. 2001;14:165–71. 11774736

[10] Topalian SL, Drake CG, Pardoll DM. Immune checkpoint blockade: a common denominator approach to cancer therapy. Cancer Cell. 2015;27:450–61. 25858804 10.1016/j.ccell.2015.03.001 PMC4400238

[11] Nishio S, Matsumoto K, Takehara K, Kawamura N, Hasegawa K, Takeshima N, et al. Pembrolizumab monotherapy in Japanese patients with advanced ovarian cancer: Subgroup analysis from the KEYNOTE-100. Cancer Sci. 2020;111:1324–32. 32012411 10.1111/cas.14340 PMC7156846

[12] Moore KN, Bookman M, Sehouli J, Miller A, Anderson C, Scambia G, et al. Atezolizumab, Bevacizumab, and chemotherapy for newly diagnosed Stage III or IV Ovarian Cancer: Placebo-controlled randomized Phase III Trial (IMagyn050/GOG 3015/ENGOT-OV39). J Clin Oncol. 2021;39:1842–55. 33891472 10.1200/JCO.21.00306 PMC8189598

[13] Lin KK, Harrell MI, Oza AM, Oaknin A, Ray-Coquard I, Tinker AV, et al. BRCA reversion mutations in circulating tumor DNA predict primary and acquired resistance to the PARP Inhibitor Rucaparib in High-Grade Ovarian Carcinoma. Cancer Discov. 2019;9:210–9. 30425037 10.1158/2159-8290.CD-18-0715

[14] Gonzalez-Martin A, Pothuri B, Vergote I, DePont Christensen R, Graybill W, Mirza MR, et al. Niraparib in patients with newly diagnosed advanced ovarian cancer. N. Engl J Med. 2019;381:2391–402. 31562799 10.1056/NEJMoa1910962

[15] Nielsen FC, van Overeem Hansen T, Sorensen CS. Hereditary breast and ovarian cancer: new genes in confined pathways. Nat Rev. 2016;16:599–612. 10.1038/nrc.2016.72 27515922

[16] Fagotti A, Ferrandina G, Fanfani F, Ercoli A, Lorusso D, Rossi M, et al. A laparoscopy-based score to predict surgical outcome in patients with advanced ovarian carcinoma: A pilot study. Ann Surg Oncol. 2006;13:1156–61. 16791447 10.1245/ASO.2006.08.021

[17] Feng J, Ding C, Qiu N, Ni X, Zhan D, Liu W, et al. Firmiana: towards a one-stop proteomic cloud platform for data processing and analysis. Nat Biotechnol. 2017;35:409–12. 28486446 10.1038/nbt.3825

[18] Ding C, Jiang J, Wei J, Liu W, Zhang W, Liu M, et al. A fast workflow for identification and quantification of proteomes. Mol Cell Proteom. 2013;12:2370–80. 10.1074/mcp.O112.025023 PMC3734592 23669031

[19] Li X, Zhang C, Gong T, Ni X, Li J, Zhan D, et al. A time-resolved multi-omic atlas of the developing mouse stomach. Nat Commun. 2018;9:4910. 30464175 10.1038/s41467-018-07463-9 PMC6249217

[20] Gaujoux R, Seoighe C. A flexible R package for nonnegative matrix factorization. BMC Bioinforma. 2010;11:367. 10.1186/1471-2105-11-367 PMC2912887 20598126

[21] Kanehisa M. Goto S. KEGG: kyoto encyclopedia of genes and genomes. Nucleic Acids Res. 2000;28:27–30. 10592173 10.1093/nar/28.1.27 PMC102409

[22] Cook SA, Comrie WA, Poli MC, Similuk M, Oler AJ, Faruqi AJ, et al. HEM1 deficiency disrupts mTORC2 and F-actin control in inherited immunodysregulatory disease. Science. 2020;369:202–7. 32647003 10.1126/science.aay5663 PMC8383235

[23] Tominaga K, Yoshimoto T, Torigoe K, Kurimoto M, Matsui K, Hada T, et al. IL-12 synergizes with IL-18 or IL-1beta for IFN-gamma production from human T cells. Int Immunol. 2000;12:151–60. 10653850 10.1093/intimm/12.2.151

[24] Kulkarni K, Yang J, Zhang Z, Barford D. Multiple factors confer specific Cdc42 and Rac protein activation by dedicator of cytokinesis (DOCK) nucleotide exchange factors. J Biol Chem. 2011;286:25341–51. 21613211 10.1074/jbc.M111.236455 PMC3137105

[25] McGinnis CS, Murrow LM, Gartner ZJ. DoubletFinder: Doublet detection in single-cell RNA sequencing data using artificial nearest neighbors. Cell Syst. 2019;8:329-+. 30954475 10.1016/j.cels.2019.03.003 PMC6853612

[26] Vazquez-Garcia I, Uhlitz F, Ceglia N, Lim JLP, Wu M, Mohibullah N, et al. Ovarian cancer mutational processes drive site-specific immune evasion. Nature. 2022;612:778–86. 36517593 10.1038/s41586-022-05496-1 PMC9771812

[27] Lambrechts D, Wauters E, Boeckx B, Aibar S, Nittner D, Burton O, et al. Phenotype molding of stromal cells in the lung tumor microenvironment. Nat Med. 2018;24:1277–89. 29988129 10.1038/s41591-018-0096-5

[28] Wang Y, Xie H, Chang X, Hu W, Li M, Li Y, et al. Single-cell dissection of the multiomic landscape of high-grade serous ovarian cancer. Cancer Res. 2022;82:3903–16 10.1158/0008-5472.CAN-21-3819 PMC9627134 35969151

[29] Zhang L, Li Z, Skrzypczynska KM, Fang Q, Zhang W, O’Brien SA, et al. Single-cell analyses inform mechanisms of myeloid-targeted therapies in colon cancer. Cell. 2020;181:442–59.e29. 32302573 10.1016/j.cell.2020.03.048

[30] Guo X, Zhang Y, Zheng L, Zheng C, Song J, Zhang Q, et al. Global characterization of T cells in non-small-cell lung cancer by single-cell sequencing. Nat Med. 2018;24:978–85. 29942094 10.1038/s41591-018-0045-3

[31] Wu TD, Madireddi S, de Almeida PE, Banchereau R, Chen YJ, Chitre AS, et al. Peripheral T cell expansion predicts tumour infiltration and clinical response. Nature. 2020;579:274–8. 32103181 10.1038/s41586-020-2056-8

[32] Ramirez PW, Famiglietti M, Sowrirajan B, DePaula-Silva AB, Rodesch C, Barker E, et al. Downmodulation of CCR7 by HIV-1 Vpu Results in Impaired Migration and Chemotactic Signaling within CD4(+) T Cells. Cell Rep. 2014;7:2019–30. 24910430 10.1016/j.celrep.2014.05.015 PMC4080720

[33] Forster R, Schubel A, Breitfeld D, Kremmer E, Renner-Muller I, Wolf E, et al. CCR7 coordinates the primary immune response by establishing functional microenvironments in secondary lymphoid organs. Cell. 1999;99:23–33. 10520991 10.1016/s0092-8674(00)80059-8

[34] Hume DA, Mabbott N, Raza S, Freeman TC. Can DCs be distinguished from macrophages by molecular signatures? Nat Immunol. 2013;14:187–9. 23416664 10.1038/ni.2516

[35] Gautier EL, Shay T, Miller J, Greter M, Jakubzick C, Ivanov S, et al. Gene-expression profiles and transcriptional regulatory pathways that underlie the identity and diversity of mouse tissue macrophages. Nat Immunol. 2012;13:1118–28. 23023392 10.1038/ni.2419 PMC3558276

[36] Corthay A, Skovseth DK, Lundin KU, Rosjo E, Omholt H, Hofgaard PO, et al. Primary antitumor immune response mediated by CD4+ T cells. Immunity. 2005;22:371–83. 15780993 10.1016/j.immuni.2005.02.003

[37] Zhang Q, He Y, Luo N, Patel SJ, Han Y, Gao R, et al. Landscape and dynamics of single immune cells in hepatocellular carcinoma. Cell. 2019;179:829–45.e20. 31675496 10.1016/j.cell.2019.10.003

[38] Condamine T, Dominguez GA, Youn JI, Kossenkov AV, Mony S, Alicea-Torres K, et al. Lectin-type oxidized LDL receptor-1 distinguishes population of human polymorphonuclear myeloid-derived suppressor cells in cancer patients. Sci Immunol. 2016;1:aaf8943. 10.1126/sciimmunol.aaf8943 PMC5391495 28417112

[39] Tcyganov E, Mastio J, Chen E, Gabrilovich DI. Plasticity of myeloid-derived suppressor cells in cancer. Curr Opin Immunol. 2018;51:76–82. 29547768 10.1016/j.coi.2018.03.009 PMC5943174

[40] Aran D, Hu Z, Butte AJ. xCell: digitally portraying the tissue cellular heterogeneity landscape. Genome Biol. 2017;18:220. 29141660 10.1186/s13059-017-1349-1 PMC5688663

[41] Cheng S, Li Z, Gao R, Xing B, Gao Y, Yang Y, et al. A pan-cancer single-cell transcriptional atlas of tumor infiltrating myeloid cells. Cell. 2021;184:792–809.e23. 33545035 10.1016/j.cell.2021.01.010

[42] Robert C, Ribas A, Schachter J, Arance A, Grob JJ, Mortier L, et al. Pembrolizumab versus ipilimumab in advanced melanoma (KEYNOTE-006): post-hoc 5-year results from an open-label, multicentre, randomised, controlled, phase 3 study. Lancet Oncol. 2019;20:1239–51. 31345627 10.1016/S1470-2045(19)30388-2

[43] Felip E, Brahmer J, Broderick S, Swanson S, Awad M, Mitsudomi T, et al. CheckMate 816: A Phase 3 trial of neoadjuvant Nivolumab Plus Ipilimumab or chemotherapy vs chemotherapy in early-stage NSCLC. J Thorac Oncol. 2018;13:S831–S2.

[44] Odunsi K. Immunotherapy in ovarian cancer. Ann Oncol 2017;28:viii1–viii7. 29232467 10.1093/annonc/mdx444 PMC5834124

[45] Wang H, Li SW, Li W, Cai HB. Elastic net-based identification of a multigene combination predicting the survival of patients with cervical cancer. Med Sci Monit: Int Med J Exp Clin Res 2019;25:10105–13. 10.12659/MSM.918393 PMC6948288 31884508

[46] Thorsson V, Gibbs DL, Brown SD, Wolf D, Bortone DS, Ou Yang TH, et al. The immune landscape of cancer. Immunity. 2019;51:411–2. 31433971 10.1016/j.immuni.2019.08.004

[47] Tang T, Cheng X, Truong B, Sun L, Yang X, Wang H. Molecular basis and therapeutic implications of CD40/CD40L immune checkpoint. Pharmacol Ther. 2021;219:107709. 33091428 10.1016/j.pharmthera.2020.107709 PMC7886970

[48] Jin SQ, Guerrero-Juarez CF, Zhang LH, Chang I, Ramos R, Kuan CH, et al. Inference and analysis of cell-cell communication using CellChat. Nat Commun. 2021;12:1088. 33597522 10.1038/s41467-021-21246-9 PMC7889871

[49] Suzuki N, Nakagawa F, Matsuoka K, Takechi T. Effect of a novel oral chemotherapeutic agent containing a combination of trifluridine, tipiracil and the novel triple angiokinase inhibitor nintedanib, on human colorectal cancer xenografts. Oncol Rep. 2016;36:3123–30. 27805254 10.3892/or.2016.5208 PMC5112602

[50] Nukatsuka M, Nakagawa F, Takechi T. Efficacy of combination chemotherapy using a novel oral chemotherapeutic agent, TAS-102, with Oxaliplatin on Human Colorectal and gastric cancer Xenografts. Anticancer Res. 2015;35:4605–15. 26254349

[51] Shitara K, Doi T, Dvorkin M, Mansoor W, Arkenau HT, Prokharau A, et al. Trifluridine/tipiracil versus placebo in patients with heavily pretreated metastatic gastric cancer (TAGS): a randomised, double-blind, placebo-controlled, phase 3 trial. Lancet Oncol. 2018;19:1437–48. 30355453 10.1016/S1470-2045(18)30739-3

[52] Mayer RJ, Van Cutsem E, Falcone A, Yoshino T, Garcia-Carbonero R, Mizunuma N, et al. Randomized trial of TAS-102 for refractory metastatic colorectal cancer. N. Engl J Med. 2015;372:1909–19. 25970050 10.1056/NEJMoa1414325

[53] Pfeiffer P, Yilmaz M, Moller S, Zitnjak D, Krogh M, Petersen LN, et al. TAS-102 with or without bevacizumab in patients with chemorefractory metastatic colorectal cancer: an investigator-initiated, open-label, randomised, phase 2 trial. Lancet Oncol. 2020;21:412–20. 31999946 10.1016/S1470-2045(19)30827-7

[54] Roviello G, Fancelli S, Gatta Michelet MR, Aprile G, Nobili S, Roviello F, et al. TAS-102 in gastric cancer: Development and perspectives of a new biochemically modulated fluroropyrimidine drug combination. Crit Rev Oncol/Hematol. 2020;152:102987. 32485527 10.1016/j.critrevonc.2020.102987

[55] Atiya HI, Orellana TJ, Wield A, Frisbie L, Coffman LG. An orthotopic mouse model of ovarian cancer using human stroma to promote metastasis. J Vis Exp. 2021;10.3791/62382. 10.3791/62382 33843939

[56] Lee S, Zhao L, Rojas C, Bateman NW, Yao H, Lara OD, et al. Molecular analysis of clinically defined subsets of high-grade serous ovarian cancer. Cell Rep. 2020;31:107502. 32294438 10.1016/j.celrep.2020.03.066 PMC7234854

[57] Pan J, Hu Y, Sun S, Chen L, Schnaubelt M, Clark D, et al. Glycoproteomics-based signatures for tumor subtyping and clinical outcome prediction of high-grade serous ovarian cancer. Nat Commun. 2020;11:6139. 33262351 10.1038/s41467-020-19976-3 PMC7708455

[58] Verhaak RGW, Tamayo P, Yang JY, Hubbard D, Zhang HL, Creighton CJ, et al. Prognostically relevant gene signatures of high-grade serous ovarian carcinoma. J Clin Investig. 2013;123:517–25. 23257362 10.1172/JCI65833 PMC3533304

[59] Lee Y, Miron A, Drapkin R, Nucci MR, Medeiros F, Saleemuddin A, et al. A candidate precursor to serous carcinoma that originates in the distal fallopian tube. J Pathol. 2007;211:26–35. 17117391 10.1002/path.2091

[60] Mehra K, Mehrad M, Ning G, Drapkin R, McKeon FD, Xian W, et al. STICS, SCOUTs and p53 signatures; a new language for pelvic serous carcinogenesis. Front Biosci. 2011;3:625–34. 10.2741/e275 21196340

[61] Falconer H, Yin L, Grönberg H, Altman D. Ovarian cancer risk after Salpingectomy: A nationwide population-based study. J Natl Cancer I. 2015;107:dju410. 10.1093/jnci/dju410 25628372

[62] Zhang L, Conejo-Garcia JR, Katsaros D, Gimotty PA, Massobrio M, Regnani G, et al. Intratumoral T cells, recurrence, and survival in epithelial ovarian cancer. N. Engl J Med. 2003;348:203–13. 12529460 10.1056/NEJMoa020177

[63] Macciò A, Gramignano G, Cherchi MC, Tanca L, Melis L, Madeddu C Role of M1-polarized tumor-associated macrophages in the prognosis of advanced ovarian cancer patients. Sci Rep. 2020;10:6096. 10.1038/s41598-020-63276-1 PMC7142107 32269279

[64] Matulonis UA, Shapira-Frommer R, Santin AD, Lisyanskaya AS, Pignata S, Vergote I, et al. Antitumor activity and safety of pembrolizumab in patients with advanced recurrent ovarian cancer: results from the phase II KEYNOTE-100 study. Ann Oncol: Off J Eur Soc Med Oncol 2019;30:1080–7. 10.1093/annonc/mdz135 31046082

[65] Gonzalez VD, Huang YW, Delgado-Gonzalez A, Chen SY, Donoso K, Sachs K, et al. High-grade serous ovarian tumor cells modulate NK cell function to create an immune-tolerant microenvironment. Cell Rep. 2021;36:109632. 34469729 10.1016/j.celrep.2021.109632 PMC8546503

[66] Bilska M, Pawlowska A, Zakrzewska E, Chudzik A, Suszczyk D, Gogacz M, et al. Th17 cells and IL-17 as novel immune targets in ovarian cancer therapy. J Oncol. 2020;2020:8797683. 32148497 10.1155/2020/8797683 PMC7054820

[67] Jimenez-Sanchez A, Memon D, Pourpe S, Veeraraghavan H, Li Y, Vargas HA, et al. Heterogeneous tumor-immune microenvironments among differentially growing metastases in an ovarian cancer patient. Cell. 2017;170:927–38 e20. 28841418 10.1016/j.cell.2017.07.025 PMC5589211

[68] Duraiswamy J, Turrini R, Minasyan A, Barras D, Crespo I, Grimm AJ, et al. Myeloid antigen-presenting cell niches sustain antitumor T cells and license PD-1 blockade via CD28 costimulation. Cancer cell. 2021;39:1623–42.e20. 34739845 10.1016/j.ccell.2021.10.008 PMC8861565

[69] Uhlitz F, Zamarin D. Rejuvenating dysfunctional T cells in ovarian cancer: CD28 is the license to kill. Cancer cell. 2021;39:1567–9. 10.1016/j.ccell.2021.10.011 34739843

[70] Karnell JL, Rieder SA, Ettinger R, Kolbeck R. Targeting the CD40-CD40L pathway in autoimmune diseases: Humoral immunity and beyond. Adv drug Deliv Rev. 2019;141:92–103. 30552917 10.1016/j.addr.2018.12.005

[71] Vonderheide RH. CD40 agonist antibodies in cancer immunotherapy. Annu Rev Med. 2020;71:47–58. 31412220 10.1146/annurev-med-062518-045435

[72] Luke JJ, Barlesi F, Chung K, Tolcher AW, Kelly K, Hollebecque A, et al. Phase I study of ABBV-428, a mesothelin-CD40 bispecific, in patients with advanced solid tumors. J Immunother Cancer. 2021;9:e002015. 10.1136/jitc-2020-002015 PMC7898862 33608377

