# Supplementary material for: Integrated proteomics and scRNA-seq analyses of ovarian cancer reveal molecular subtype-associated cell landscapes and immunotherapy targets
Source: Br J Cancer. 2024 Nov 15;132(1):111–25. doi: 10.1038/s41416-024-02894-2 (PMC11723995; doi:10.1038/s41416-024-02894-2)
Supplement: Supplementary file 1 — supplementary information [file 41416_2024_2894_MOESM1_ESM.docx]

**Integrated proteomics and scRNA-seq analyses of ovarian cancer reveal molecular subtype-associated cell landscapes and immunotherapy targets**

Rong Tan^#,*^, Ming Wen^#^, Wenqing Yang, Dongdong Zhan, Nairen Zheng, Mingwei Liu, Fang Zhu, Xiaodan Chen, Meng, Wang, Siyu Yang, Bin Xie, Qiongqiong He, Kai Yuan, Lunquan Sun, Yi Wang^*^, Jun Qin^*^, and Yu Zhang^*^

**Methods**

**Additional Clinical Data**

A total of 183 specimens from 82 patients, including 29 normal fallopian tubes and 154 tumor samples. 29 normal fallopian tubes were provided by patients including 21 high-grade serous carcinoma (HGSC), 4 ovarian endometrioid carcinoma (OEC), 1 mucinous ovarian carcinoma (MOC), 1 ovarian clear cell carcinoma (OCCC), 1 mixed cell carcinoma and 1 colorectal ovary metastases carcinoma. Among those 29 patients, 16 participants only contributed samples of normal fallopian tubes and 13 participants contributed paired tumor samples.

154 OC tumor samples were from 66 OC patients (22 patients with only primary tumor lesions, 44 patients with primary tumor lesions and their omental and peritoneal metastatic lesions) including 137 HGSCs from 49 OC patients (5 samples from patients with only primary tumor, 132 samples from 44 patients with paired primary tumor, omental, and peritoneal metastatic lesions) and 17 other histological subtypes(3 clear cell carcinoma, 8 low-grade serous carcinomas, 4 mucinous carcinomas, and 2 adenocarcinomas from 17 OC patients. 13 of 69 patients have paired normal fallopian tubes. There were 22 patients from stage I and stage II and 44 patients from stage III and stage IV. 44 patients developed metastatic tumors at sites other than the primary location, and 14 patients had lymph node metastasis, confirmed by histopathological analysis.8 fresh primary tumor samples were collected from ovarian cancer patients who underwent primary debulking surgeries including 7 HGSCs and 1 clear cell carcinoma.

Hematoxylin and eosin (H&E) stained sections from each sample were subjected to independent pathology review to confirm that the tumor specimen was histologically consistent with the submitted diagnosis. The detailed sample information is showed in Table S1A, S1B.

Women suspected of ovarian cancer would receive contrast-enhanced computerized tomography (CT) scans and serologic tests such as CA125. Test results were subsequently reviewed by the tumor board, including gynecology oncologist, radiologist and surgeon, to discuss whether the patient receives neoadjuvant chemotherapy or primary debulking surgery after the biopsy.

If the case could not be determined under the tumor board discussion, the Fagotti’s Score([63](#_ENREF_63)) would be evaluated through the laparoscopy. The patients with Fagotti’s score higher than 8 would receive 2 or 3 cycles of neoadjuvant chemotherapy and then re-evaluate the possibility of optimal debulking surgery.

Cases were staged according to the 1988 International Federation of Gynecology and Obstetrics (FIGO) staging system and underwent close observation or platinum-based chemotherapy after surgery in strict accordance with the National Comprehensive Cancer Network guidelines (NCCN). Residual disease and volume of Ascites were recorded after debulking surgery.

HE-stained slides were examined and evaluated independently by two experienced pathologists and information regarding tumor histological subtype, degree of differentiation, FIGO staging, and tumor purity was provided. Immunohistochemical staining of all tumor specimens showed PAX8 (+), WT1 (+), CA125 (+), CK7 (+), CDX-2 (-), CK20 (-), CEA (-) refer to diagnose ovary as primary site. All tumor samples used in this study had tumor purity >= 50%. Once the diagnosis of ovarian cancer was confirmed, genetic risk evaluation was recommended for each patient.

Patients were followed up every three months for at least 3 years. Progression-Free-Survival (PFS, defined as period from last postoperative chemotherapy to recurrence indicated by any serological or radiographic signs) and overall survival (OS, defined as period from diagnosis to death) were calculated. Patients defined as complete remission or relapse ≥12 months after completing prior chemotherapy were considered platinum-sensitive diseases. Patients defined as complete remission or relapse between 6 ~ 12 months after completing prior chemotherapy were considered partially platinum-sensitive disease. Patients defined as progression on primary therapy, stable or persistent disease or complete remission and relapse <6 months after completing the chemotherapy were considered platinum-resistant disease.

In this study, 32 of the 66 OC patients had complete gross resection of disease (R0), 22 had microscopic residue (R1), and 12 had gross tumor residue (>R1). 3 of 66 patients didn’t receive chemotherapy due to pathology and early stage, while 63 patients received platinum-based chemotherapy. Among them, 9 patients were considered platinum-resistant. 10 patients in our cohort accepted the recommendation for genetic testing, and only Patient #17 got BRCA1 germline mutation and Patient #42 got BRCA2 germline mutation. All the detailed clinical information and therapies of patients in this study were included in Table S1A.

**Protein extraction and tryptic digestion for Mass spectrometry**

Tissues were cut into small pieces and placed in a microcentrifuge tube. Wash tissues with PBS and centrifuge tissue at 500 g for 5 minutes. Cell pellets were lysed in 1% sodium deoxycholate, 10 mM Tris (2-carboxyethyl) phosphine, 40 mM 2-chloroacetamide and 100 mM Tris–HCl pH 8.5 at 95 °C for 5 min and by 5 min sonication (3s on and 3s off, amplitude 25%). After 16,000 g centrifugation at 4 °C for 10 min, the supernatants were reserved as whole tissue extract. 100 μg proteins in the cleared lysate were digested overnight with 1:50 trypsin (Promega, USA) at 37 °C. The next day, digestion was stopped by adding 1% formic acid. Precipitated sodium deoxycholate was removed by 10 min 16,000 g centrifugation at 4 °C and peptides in supernatant were desalted on C18 StageTips. Desalted peptides were vacuum-dried and stored at –80 °C until subsequent LC-MS/MS analysis.

**Tissue dissociation and preparation of single-cell suspensions**

Single cells were collected from primary tumor tissues freshly through surgical operation. Place a sterile RNase-free culture dish containing an appropriate amount of calcium-free and magnesium-free 1x PBS on ice, the tissue was transferred into the culture dish and cut into 0.5 mm^2^ pieces, the tissues were washed with 1 × PBS, and removed as many non-purpose tissues as possible such as blood stains and fatty layers.

The tissues were dissociated into single cells in dissociation solution (0.35% collagenase IV5, 2 mg/ml papain, 120 Units/ml DNase I) in 37°C water bath with shaking for 20 min at 100 rpm. Digestion was terminated with 1x PBS containing 10% fetal bovine serum (FBS), then pipetting 5-10 times with a Pasteur pipette. The resulting cell suspension was filtered by passing through a 70 mm stacked cell strainer and centrifuged at 300 g for 5 min at 4℃. The cell pellet was resuspended in 100μl 1x PBS (0.04% BSA) and added with 1 ml 1x red blood cell lysis buffer (MACS 130-094-183, 10x) and incubated on ice for 2-10 min to lyse the remaining red blood cells. After incubation, the suspension was centrifuged at 300 g for 5 min at room temperature. The suspension was resuspended in 100 μl Dead Cell Removal MicroBeads (MACS 130-090-101) and removed dead cells using Miltenyi ® Dead Cell Removal Kit (MACS 130-090-101). Then the suspension was resuspended in 1x PBS (0.04% BSA) and centrifuged at 300 g for 3 min at 4°C (repeat twice). The cell pellet was resuspended in 50µl of 1x PBS (0.04% BSA). The overall cell viability was confirmed by trypan blue exclusion, which needed to be above 85%, single cell suspensions were counted using a hemocytometer/ Countess II Automated Cell Counter and concentration adjusted to 700-1200 cells/μl.

**FACS (Flow cytometry) analysis of patients’ tumor samples**

The fresh collected surgical tumor tissues were washed with cold PBS and then cut into small pieces but without disrupting the connection between pieces. Next, put the tumor tissues into a 50 ml tube containing 10 ml washing buffer (10% FBS, 65 mM DTT in PBS) and shake at 200 rpm for 15 min at 37C°. Wash with 10 ml PBS to remove the DTT thoroughly before the tumor tissues were gently sliced into 1–2 mm pieces using a scalpel and dissociated in 1640 medium supplemented with 0.3 mg/ml collagenase type I (17104-019; Sigma-Aldrich) and 0.1 mg/ml collagenase type IV (17104-019; Sigma), 0.1 mg/ml DNase I (H3506; Sigma), and 10% FBS for 1 h at 37°C, shaking at 200 rpm for mixing. The resultant cell suspension was then filtered through 70-µm cell strainers. Cell suspensions collected as described above were washed with PBS and stained with Zombie UV viability dye (Bio-Legend 423101) and then washed with FACS buffer (1x PBS, 2% FBS, 2mM EDTA). The cells were then stained with multiple fluorophore-conjugated cell-surface antibodies (BV421-CD40 Bio-legend 334331, APC-CD68 Bio-legend 333809, PerCP/Cyanine5.5 CD11c Bio-legend 301623, APC-Cy7 CD45 Bio-legend 304014, FITC CD3 Bio-legend 317305, PE-Cy7 CD8 Bio-legend 344711, PE CD19 Bio-legend 302207). These antibodies were arranged into different panels, and compensation settings were established before running the stained cells on a Fortessa X-20 (BD Biosciences, San Jose, CA, USA). Storage events were gated on populations of interest. Flow data were analyzed using Flow Jo v.10 (FlowJo, Ashland, OR, USA).

**Plasmid constructions**

pLVX-Flag-SBP-tagged TYMP was constructed by inserting genes cloned from the cDNA of A2780 cells into the pLVX-Flag-SBP vector using Sal I (Takara, #1166A) and Not I (Takara, #1080A) restriction sites.

**Patient-derived xenograft (PDX)**

The animal experiments were approved by the Animal Ethics Committee of Xiangya Hospital Central South University. Immunodeficient SCID-NOD mice were obtained from Hunan SJA Laboratory Animal Co.,LTD and maintained under pathogen-free conditions in-house, under Central South University Institutional Animal Care and Use Committee approved protocols. Tumor was obtained from ovarian cancer debulking surgeries without neoadjuvant chemotherapy. Fresh tumor fragments engrafted 3 pieces (2–3 mm^3^ each) subcutaneously (s.c.) into the flanks of the back of SCID-NOD female mice. Mice were observed for a maximum of 120 d and maintained under sterile and controlled conditions (26 °C, 50% relative humidity, 12 h light-dark cycle, autoclaved food and bedding). Tumor growth was measured in 2 dimensions with a caliper. Tumor volumes (TV) were determined by the formula: TV = (width² x length) x 0.5. Tumors were routinely passed at TV = 1 cm³. Groups of 4 mice were randomized to receive either solvent as control or 150mg/kg TAS102 drugs orally (S8539, selleck) as indicated in Figure 6D. At first, the dose of TAS102 was implemented according to the recommended concentration in the literature ([64](#_ENREF_64), [65](#_ENREF_65)) and website of Selleck. However, after 3 consecutive days of drug feeding, the mice showed significant weight loss (Figure S6B). So we reduce the dose of the TAS102 to 150 mg/kg per two days. Treatment was started at a tumor size of approx. 0.1 cm³ (80 - 120 mm³). Xenograft material was snap-frozen and stored at -80 °C or processed to formalin-fixed, paraffin-embedded (FFPE) blocks.

**Orthotopic mouse model of ID8 ovarian cancer cells**

Female C57BL/6J mice (6–8 weeks old, Hunan SJA Laboratory Animal Co., Ltd) were housed under pathogen-free conditions according to protocols approved by the Central South University Institutional Animal Care and Use Committee. An orthotopic ovarian cancer model was generated via microsurgery by injecting 0.5 × 10^6 ID8 TYMP^+^ Luc^+^or ID8 Vector Luc^+^ cells into the bursa of the left ovary. One week post-injection, groups of 5 mice were randomized to receive either 0.5% hydroxypropyl methylcellulose (HPMC) as a control or 150 mg/kg TAS102 orally (S8539, Selleck) as outlined in Figure S6B. Mice were sacrificed when HPMC-treated mice developed severe ascites or TAS102-treated mice exhibited significant weight loss and became moribund, at which point tumor counts were performed. Overall condition and body weight were monitored every three days, and tumor burden was assessed weekly. Bioluminescence signals from ffLuc+ cells were detected using the In Vivo Imaging System (IVIS, PerkinElmer) following intraperitoneal injection of 150 mg/kg D-luciferin (Beyotime, ST196-500mg) in PBS, with data analyzed using Living Image V.4.3.1 software (PerkinElmer). Mice were sacrificed upon reaching the humane endpoints as previously described ([66](#_ENREF_66), [67](#_ENREF_67)).

**Immunohistochemistry**

Tumor sections were embedded in paraffin and mounted on plus slides. The slides were baked in 68℃ for 1h and Deparaffinize slides in xylene for 2 times, 5 min each, then hydration by gradient descent ethyl alcohol (100%, 95%, 90%, 85%, 80%, 70%). After hydration, citrate buffer to perform antigen retrieval and then blocked endogenous peroxidase activity by 3% H_2_O_2_. Tumor sections were blocked by 5%BSA in PBS and incubated 20min at room temperature. Washed slides with PBS 3 times, 3 min each. Primary antibody dilution (Flag (T1804, Sigma, 1:500), TYMP (MA5-13542, Invitrogen, 1:200, RRID: AB_10985215), Caspase 3 (19677-1-AP, proteintech, 1:500), Ki67 (MAB-0672, MXB biotechnologies), Ki67 (ab15580, abcam, 1:500) to the sections on the slides and incubated at 4℃ overnight. The next day applied diluted Sav-HRP conjugates were incubated for 20 min at RT, washed 2 times with PBS and revealed the color of antibody staining by DAB substrate solution, washed slides with PBS and counterstain slides by immersed sides in Hematoxylin for 30s. Slides were then rinsed in running water for 10 min. Dehydrate the tissue slides through gradient rise ethyl alcohol (70%, 80%, 85%, 90%, 95%, 100%). Finally, cleared slides by xylene and coverslip mounting were used with neutral resins.

**Protein identification and quantification**

MS raw files were searched against the National Center for Biotechnology Information (NCBI) Ref-seq human proteome database in Firmiana implemented with the Mascot search engine (Matrix Science, version 2.3.01)([68](#_ENREF_68)). The following search parameters were used: the mass tolerances were 20 ppm for precursor ions and 0.5 Da for product ions. Protein identification and label-free, intensity-ased absolute quantification (iBAQ) were carried out as previously described([69](#_ENREF_69)). The fraction of total (FOT), calculated by a protein's iBAQ divided by total iBAQ of all identified proteins in one experiment, was used to represent the normalized abundance of a protein across experiments. For easy representation, the FOT was then multiplied by 10^5^ to obtain iFOT ([70](#_ENREF_70)). Missing values were substituted with zeros.

**Differential proteome analysis & PCA**

Differential ANOVA proteome analyses were performed based on proteome profile comparison each kind of tissue (fallopian, ovary, omentum and peritoneum) and 4 Proteomic subtypes. The proteins with P-values in the ANOVA test lower than 0.05 were determined as differential proteins and then used for PCA analysis by R packages preprocessor.

The differential expression of proteins between two groups (CD40 high and CD40 low) was analyzed by Wilcoxon rank sum test. The proteins were determined as significant signature proteins when the fold changes comparing two groups were higher than two and P-values were lower than 0.05.

**Single-cell RNA-Seq data processing**

The Cell Ranger toolkit (version 2.1.0) provided by 10X Genomics was applied to align reads and generate the gene-cell unique molecular identifier (UMI) matrix, using the reference genome GRCh38. The data was filtered to include genes that were expressed in at least 3 cells and cells that expressed at least 200 genes, not more than 8000 genes and less than 5% of mitochondrial transcripts. We used a relatively high threshold to ensure that we have filtered out the most of barcodes associated with empty partitions or doublet cells. Cells with unusually high detection rates (>5%) of mitochondrial gene expression were also excluded.

**Cell doublet detection and removal**

We applied DoubletFinder([72](#_ENREF_72)) in human myeloid subset to identify artifactual libraries generated from two or more cells, where they entered the same microfluidic droplet and were labeled with barcodes. The doublet score for each single cell and the threshold based on the bimodal distribution were calculated using default parameters. We used a cluster-level approach to remove doublet clusters containing a large number of potential doublet cells. Specifically, we removed the CD14+ IL32+ cluster with a large fraction of potential monocyte-NK cell doublets, which expressed both monocyte signature genes (CD14, S100A8, S100A9, S100A12) and cytotoxic signature genes (IL32, NKG7, GZMA, GZMB) (data not shown).

**Dimension reduction and unsupervised clustering**

Single-cell data were processed for dimension reduction and unsupervised graph-based clustering algorithm following the workflow in Seurat (version 4.1.1). In brief, 2,000 highly-variable genes were selected for downstream analysis by using FindVariableFeatures function with the ‘vst’ method and parameter ‘nfeatures = 2000’. Then, a principal component analysis (PCA) was performed on about 2000 variable genes. We used the function FindClusters on 15-25 PCs with resolution 0.2-1 to perform the first-round cluster and annotated each cluster by known markers. We identified six major cell types, including 3 immune cell types (T cells, B cells and myeloid cells) and 3 non-immune cell types (epithelial cells, endothelial cells, and the second-round clustering was performed to identify clusters within the major cell types aforementioned according to the same range of parameters.

For visualization, the dimensionality of each dataset was further reduced using Uniform Manifold Approximation and Projection (UMAP) implemented with Seurat functions RunUMAP. The PCs used to calculate the embedding were as the same as those used for clustering. To integrate single cells for correcting the patient effects, we utilized integrated analysis (CCA) by the Seurat v3 function IntegrateData. Importantly, the new integrated matrix was obtained and was used only for visualization, instead of clustering or any other downstream analyses.

**Identification of signature genes and Gene set enrichment analysis**

We identified differentially expressed genes (DEGs) based on analysis of FindAllMarkers function in Seurat, and used wilcox.test in R to evaluate the significance of each gene. Genes with adjusted P-value less than 0.05 and the log2 fold change (log2 FC) higher than 0.25 were considered as differentially expressed genes. To calculate pseudo-cell type distributions for the 4 proteomic subtypes based on protein expression (Figure 3F-H), We selected cell type specific genes from the 8 sets of scRNAseq data and refined cell type signature proteins for each subtype from differentially expressed proteins based on proteomics data (anova test p<0.05, fold-change > 2 between highest expressing/2nd highest expressing subtype) in Xiangya OC, CPTAC 2016 or 2023 OC cohorts.

Gene set enrichment analysis was performed on the results of the differential gene expression analysis using the clusterProfile package (version 4.2.2). Gene set score was calculated with “Seurat::AddModuleScore” function.

**Cell-cell interaction analysis**

We used Cellchat ([73](#_ENREF_73)) to infer cell-cell interaction between immune cell subsets. The potential interaction strength between two cell subsets was predicted based on the expression of ligand-receptor pairs. The enriched ligand-receptor interactions between two cell subsets were calculated based on a permutation test.

We extracted significant ligand-receptor pairs with P-value < 0.05.

**Code availability**

Specific code will be made available upon request (without restrictions) to biowming@csu.edu.cn.

**Supplementary figure legends**

**Figure S1. Proteomics-based NMF molecular subtyping**

1. Venn diagrams depicting overlapped patients collected in the primary ovary, omentum, peritoneum tumor sites and fallopian tubes. **B**. The clinical information for samples, including histological types, pathological types, and analyses including proteomic and single RNA-seq. A total of 183 samples were collected from 82 patients, including 154 tumor tissues and 29 normal fallopian tissues. The 154 tumor tissues were collected from 66 OC patients, mainly consisting of 22 primary tumors (5 HGSCs+17 other EOCs) from early-stage patients and 132 tumors from 44 advanced-stage patients (containing paired primary, peritoneum metastasis, and omentum metastasis tumor sites). The 29 normal fallopian tissues were made up of normal tubes from 13 patients overlapping with tumor donors and 16 other participants only contributed samples of normal tubes. **C.** The accumulation of gene products identified in each sample. **D.** Dynamic ranges of the proteomes measured. The relative abundance of the proteins was indicated by iFOT, which was obtained by multiplied 10^5^ of the fractions of total (FOT). **E.** Algorithm for sample quality control and proteins selected for NMF subtyping analysis for Xiangya OC proteomics. **F.** NMF Silhouette width plots for 4 subtypes drawn by CancerSubtypes package. **G.** NMF subtyping for Xiangya 183 samples. **H.** The K-M curves of OS by NMF 3 (left) and 5 (right) subtypes of all Xiangya OC patients. **I.** Algorithm for sample quality control and proteins used for NMF subtyping analysis for 2016 CPTAC OC validation cohort. **J.** NMF proteomic subtyping for 2016 CPTAC OC validation cohort. **K.** Dotplot showing GO-BP (biological progress) analysis of feature proteins for Proteomic subtypes of CPTAC 2016 OC validation cohort. **L-M.** NMF proteomic subtyping for 2023 OC validation cohort. **N.** The K-M curves of PFS (left) or OS (right) by NMF 4 subtypes of Xiangya OC patients with HGSC. The tables below show the numbers of patients in follow-up at the year as indicated.

**Figure S2. Proteomics-based molecular subtyping associated with clinical outcomes**

**A.** The association of proteomic subtypes with 3 clinical variables. Kruskal-Wallis test was used for continuous variables: ascites (ml, the volume of fluid collected in spaces within patients’ abdomen before debulking surgeries or neoadjuvant chemotherapy); HE4 (pmol/L, the serum HE4 detected before debulking surgical or neoadjuvant chemotherapy). Fisher’s exact tests were used for other categorical variables**. B.** The distribution of samples with ascites in 4 subtypes (*, P < 0.05, two-sided, unpaired t test). **C.** The distribution of samples with different platinum responses in 4 Proteomic subtypes of 2023 corhort.

**Figure S3. Single cell RNA-seq reveals cell ecosystem of the proteomic subtypes**

**A.** Algorithm of 183+1 method was employed for NMF subtyping analysis for 8 validation OC samples. Briefly, a new sample was put into the original cohort of 183 samples one by one with the same NMF quality controls as indicated. After re-subtyping, the subtype of a new sample could be obtained. **B.** Clustered heatmap of RNA-seq copy number profiles estimated by inferCNV in epithelial, fibroblasts and endothelial cells. Barplot (right) showing the CNV score of indicated cells. **C.** Ratio of 6 cell types relative to the total cell count per group (the total cell count was scaled to 1.

**Figure S4. GZMK CD8+ T cell and MRC1 TAM like macrophages consist of highly infiltrative immune cells in the immune infiltrating subtype**

**A.** The percentages of patients with lymph node excision and metastasis in each NMF proteomics subtype. **B.** UMAP plot showing the distribution of 8 scRNA-seq samples colored by immune infiltrating and non-immune proteomic subtypes (left). **C.** Ratio of CD45+ or CD45- cell types relative to the total cells in immune infiltrating and non-immune subtypes (left), the total cell count was scaled to 1. Barplot showing the percentage of CD45+ or CD45- cell types normalized to total cells in immune infiltrating and non-immune subtypes (right). Two-sided Wilcoxon test, *, p <0.05, error bar indicates ±SEM. **D.** Ratio of CD45+ Cell type relative to the total cell count per sample, the total cell count was scaled to 1 **E.** Flow cytometry analysis showing representative lymphocytes in tumor samples of immune infiltrating subtype (red) and non-immune (blue) subtypes. **F.** UMAP plot showing the expression levels of selected marker genes. **G.** Heatmap shows the expression signature of their top marker genes for 7 T cell subclusters. **H.** Ratio of T cell subclusters relative to the total T cell count in immune infiltrating subtype samples. **I.** Heatmap showing the expression signature of their top marker genes for 7 Myeloids subclusters. **J.** Ratio of myeloid cell subclusters relative to the total myeloid cell count in immune cells. The total myeloid cell count was scaled to 1. The figure notes are the same as in I. **K.** Bubble heatmap showing gene signature score of TAMs, MDSCs, M1_score, M2_score, Angiogenesis and Phagocytosis in myeloid subclusters as indicated. Bubble size represents the proportion of cells with enrichment gene score > 1. The color of the circle represents the enrichment gene scores. **L.** Bubble plot showing the GO-BP (biological progress) analysis of differentially expressed genes in Myloids between C2 and C1/C3/C4 analyzed with scRNAseq. **M.** The interaction strength and numbers of immune cells in immune infiltrating and non-immune subtypes inferred by cellchat. Bubble size represents the interaction strength. The color of the circle represents the interaction numbers. **N.** Number of significant ligand-receptor pairs between T1 CD8+ GAMK and other immune cell populations. The edge width is proportional to the indicated number of ligand-receptor pairs.

**Figure S5. CD40 suggests a potential therapeutic strategy in the immune infiltrating subtype**

**A.** The association of CD40 protein expression with PFS within patients in subtype C1, C3, C4 or total C1/C3/C4 of Xiangya OC proteomics. Log-rank test. **B.** The association of CD40 protein expression with PFS within patients in all patients, or in C1, C2, C3 subtypes in CPTAC 2016 OC proteomic validation cohort. **C.** CD40 protein abundance in each NMF proteomics subtype in 2016 CPTAC OC proteomic validation cohort (**, P < 0.01, *, P < 0.05, two-sided, unpaired t test). **D.** Representative multispectral immunofluorescent images of CD40, CD40L, CD4, CD8, CD68 and DAPI in patients’ tumor samples of C2 and C1 subtypes. Scale bar, 50 μm.

**Figure S6. Druggable candidates particularly TAS102 would be a new selective chemotherapy for ovarian cancer**

**A.** The protein abundance of TYMP in tumor and fallopian tissues in Xiangya OC proteomics (***, P < 0.001, *, P < 0.05, two-sided, unpaired t test). **B.** The association of TYMP protein expression with OS within patients in HGSC of non-immune subtype in Xiangya OC proteomics. Log-rank test. **C.** The association of TYMP protein expression with OS within patients in Immune subtype C2 patients in CPTAC 2016 OC proteomic validation cohort. **D.** The association of TYMP protein expression with OS within patients in HGSC of Immune subtype in Xiangya OC proteomics. Log-rank test. **E.** The proteomic protein abundance of TYMP in each sample. **F.** The tumor weight was measured at the end of the treatments (n = 4, Error bars represent SEM, ***, P < 0.001, two-sided, unpaired t-test). **G.** The weights of NOD-SCID mice measured at time points after TAS102 treatments. **H.** Barplot showing the expression of *TYMP* across 6 cell subpopulations in immune infiltrating and non-immune subtypes (***, P < 0.001, **, P < 0.01, *, P < 0.05, Wilcox test). The number of each cell subpopulation was shown below the figure. **I.** Barplot showing the expression of *TYMP* across CNVscore high and CNVscore low subpopulations in immune infiltrating and non-immune subtypes. **J.** Schematic diagram of ID8 ovarian cancer cells orthotopic mice model. Time schedule and concentration (150 mg per kg, oral) of treated TAS102 as indicated (black arrow). **K.** The weights of ID8 mice were measured at time points after TAS102 treatments. **L.** Representative images of IHC staining with cell proliferation marker HE, Flag and Ki67 in tumor samples derived from ID8 mice. Scale bar, 100 μm.
