# Supplementary figures and images for: Integrated proteomics and scRNA-seq analyses of ovarian cancer reveal molecular subtype-associated cell landscapes and immunotherapy targets
Source: Br J Cancer. 2024 Nov 15;132(1):111–25. doi: 10.1038/s41416-024-02894-2 (PMC11723995; doi:10.1038/s41416-024-02894-2)

L

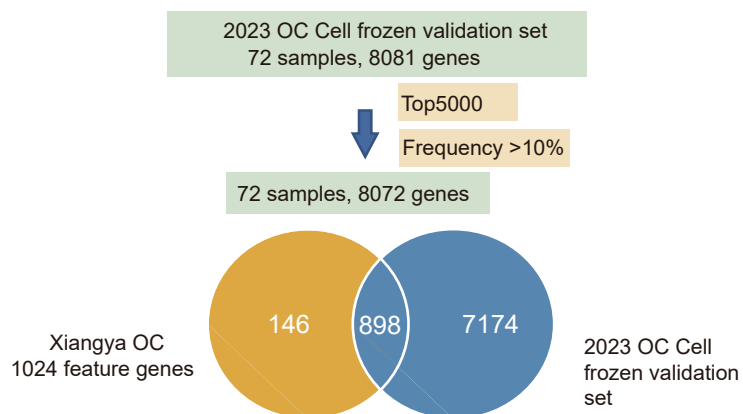

M

## NMF subtyping

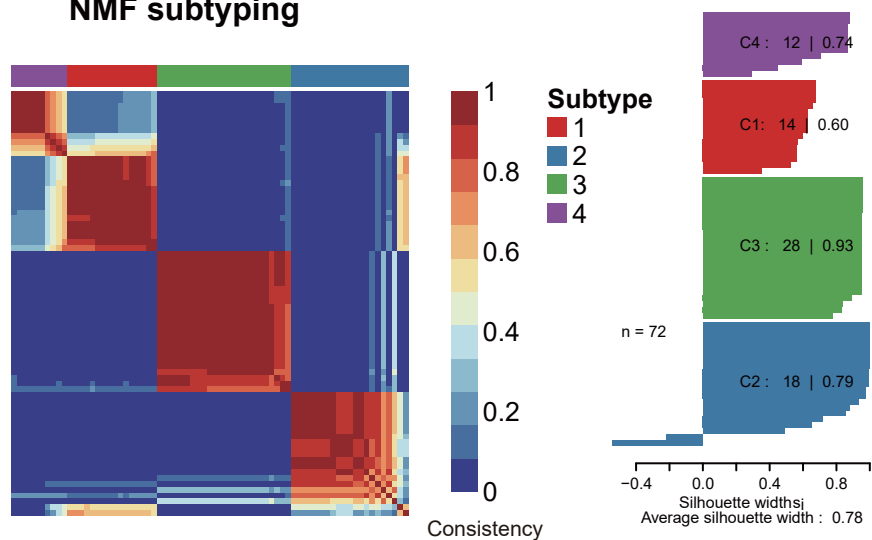

N

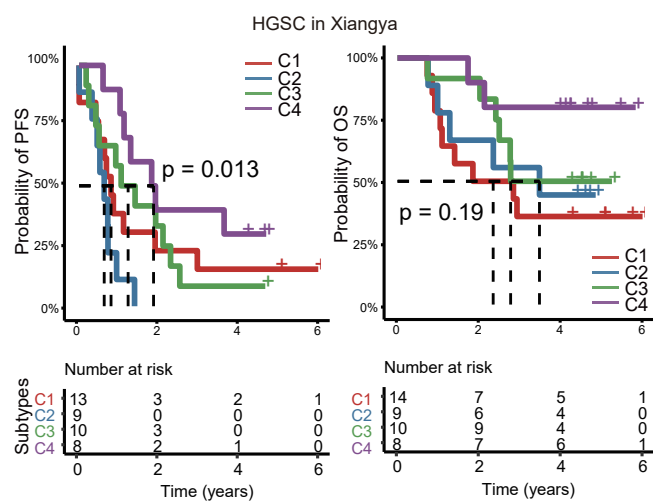

Supplement: Supplementary file 2 — Figure S1 [file 41416_2024_2894_MOESM2_ESM.pdf]

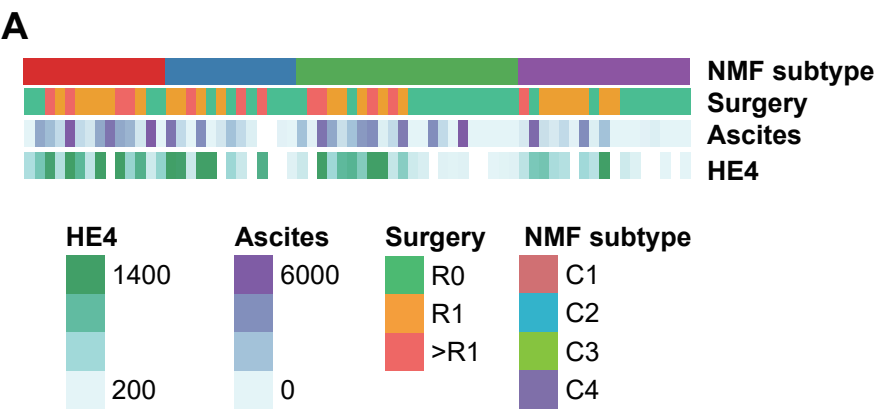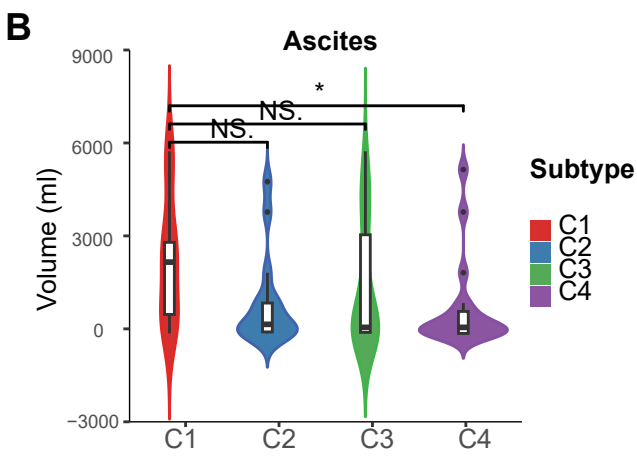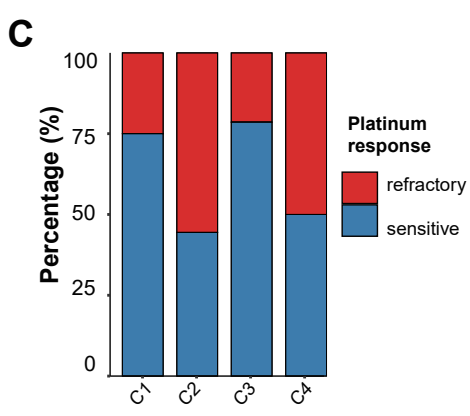

Supplement: Supplementary file 3 — Figure S2 [file 41416_2024_2894_MOESM3_ESM.pdf]

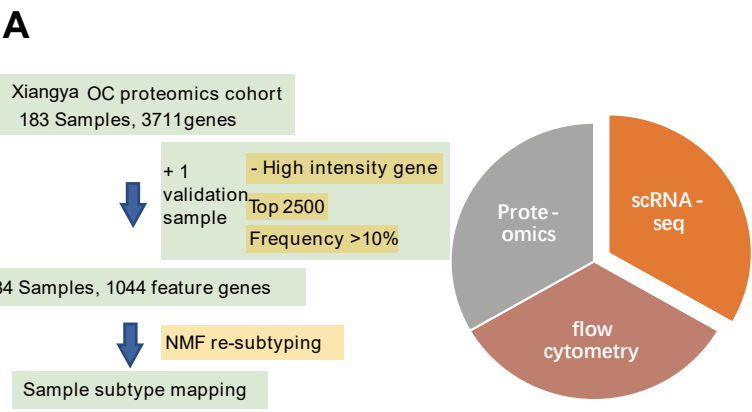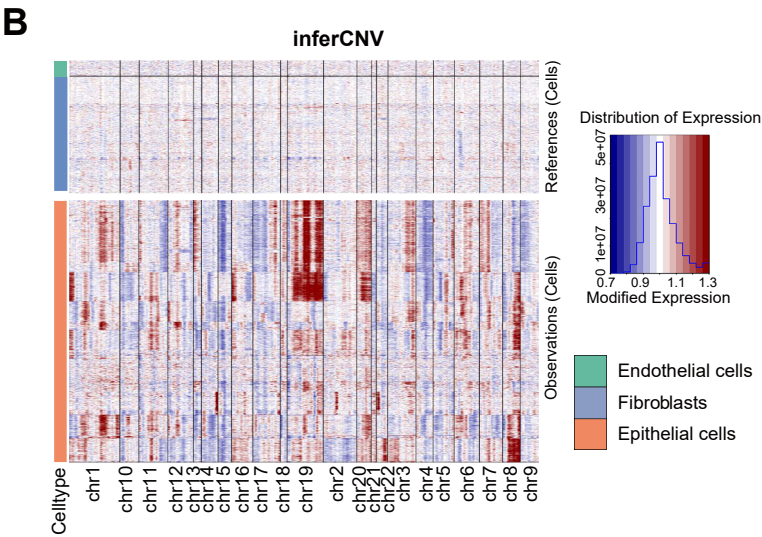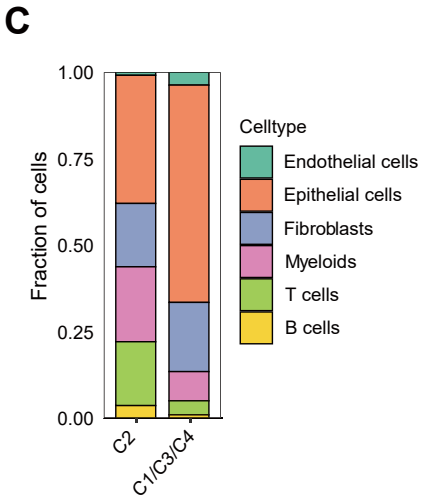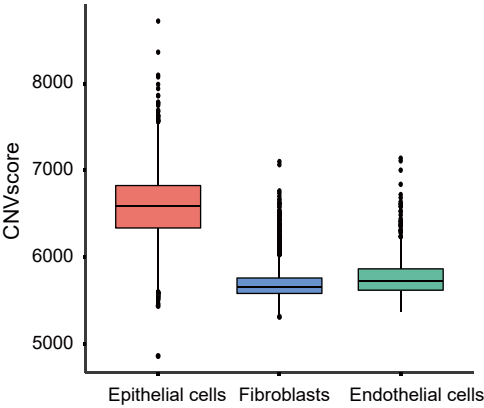

Supplement: Supplementary file 4 — Figure S3 [file 41416_2024_2894_MOESM4_ESM.pdf]

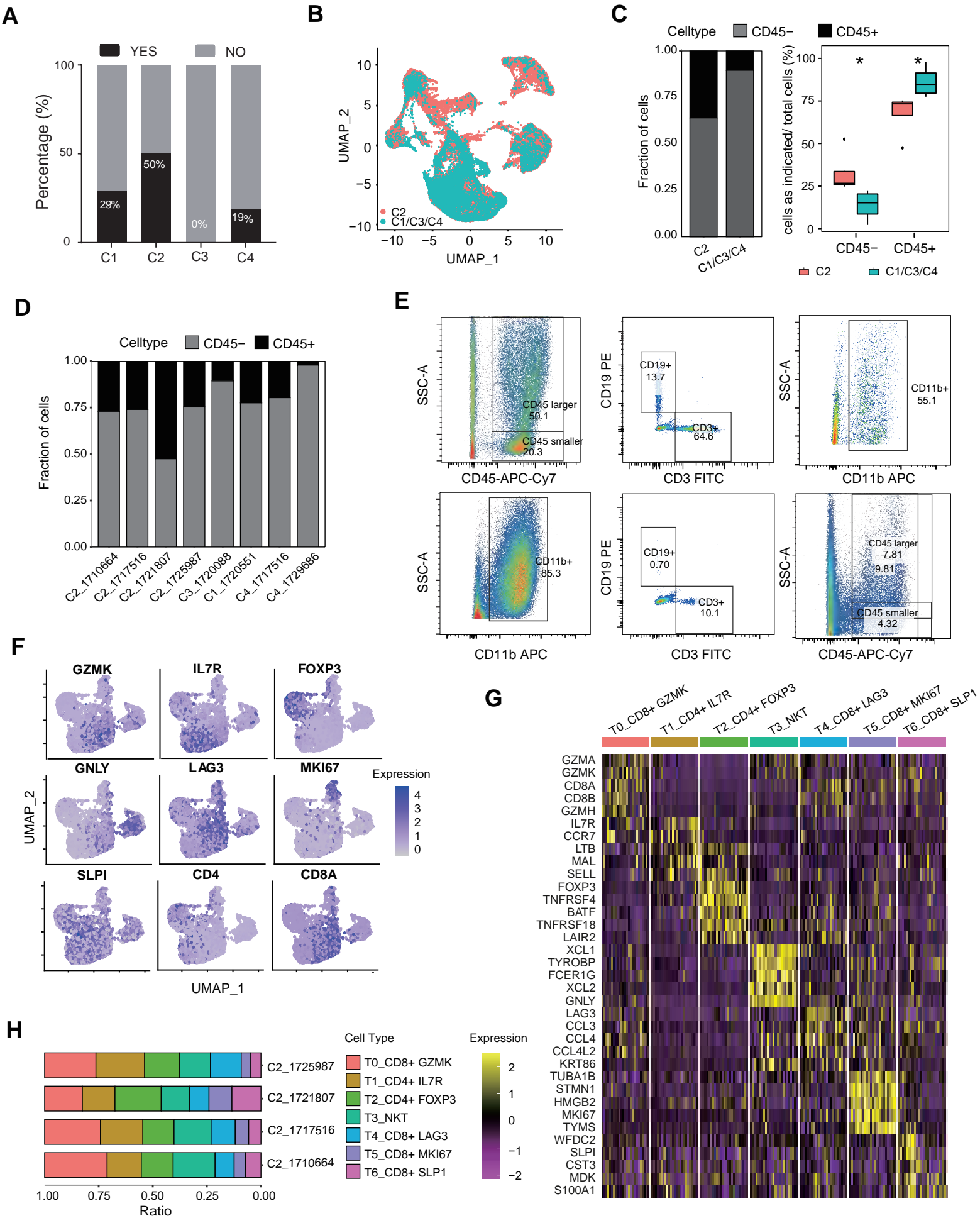

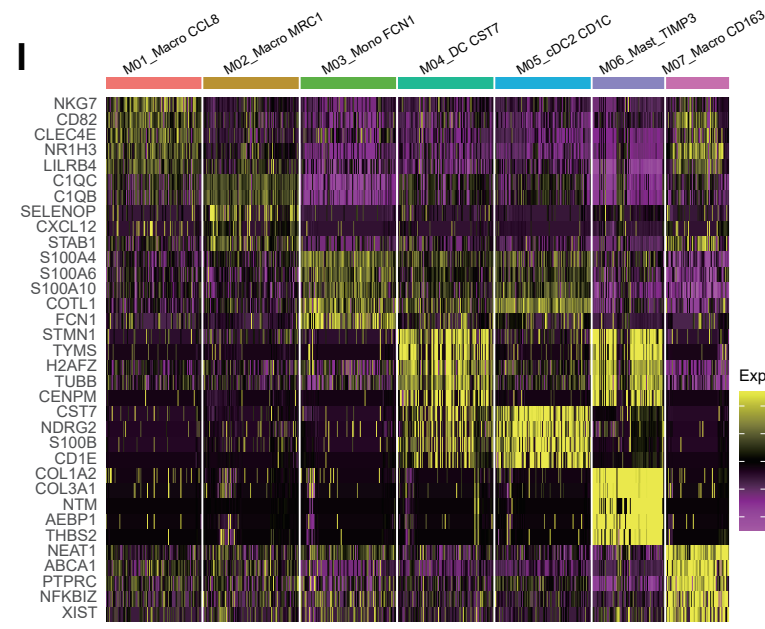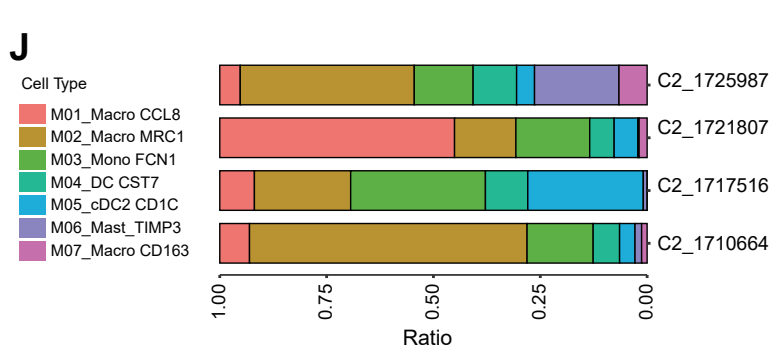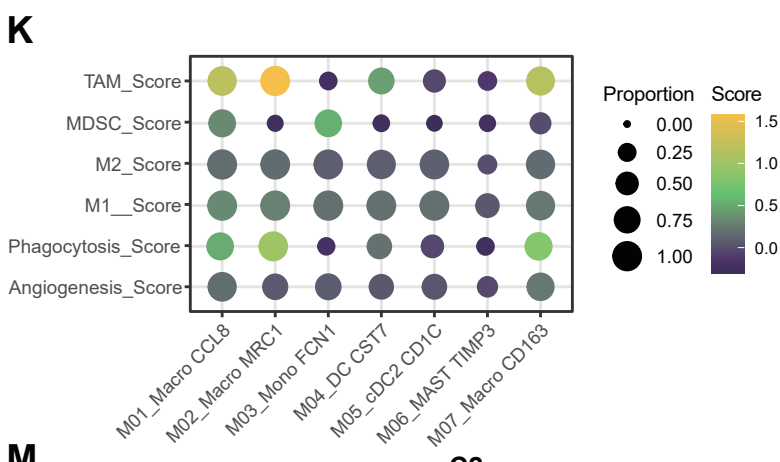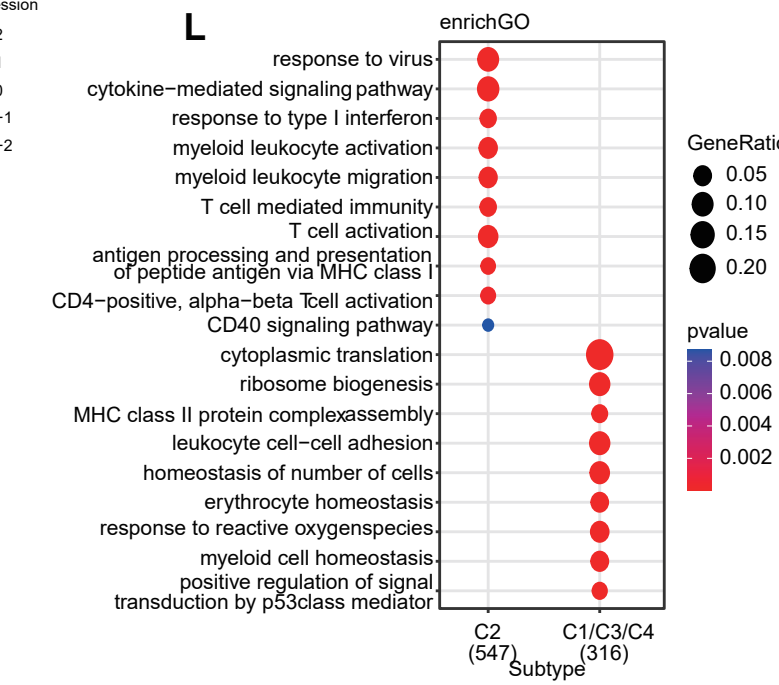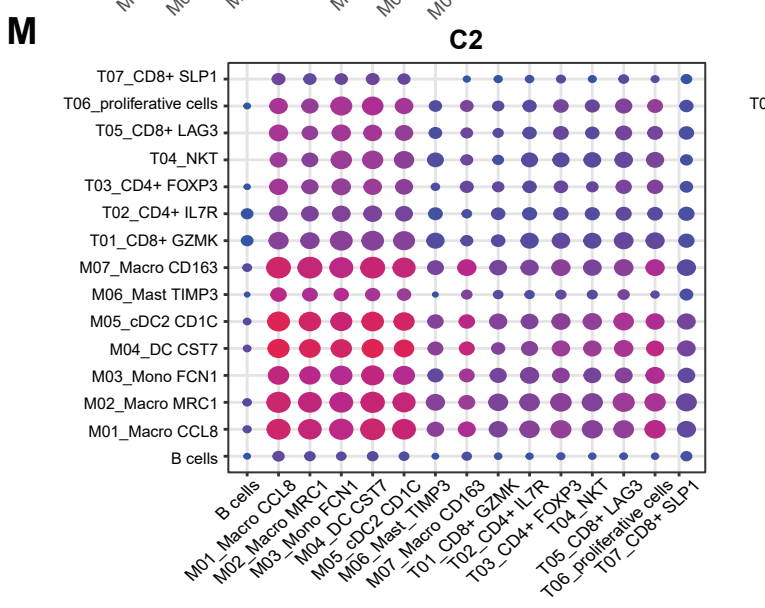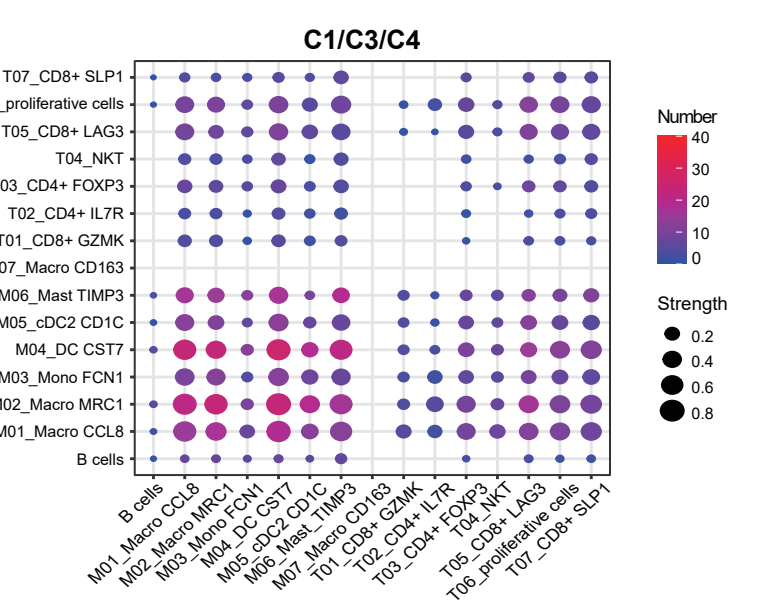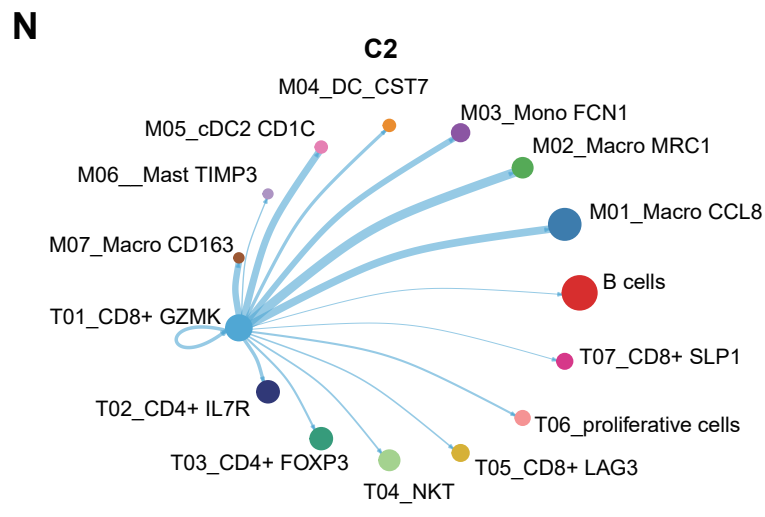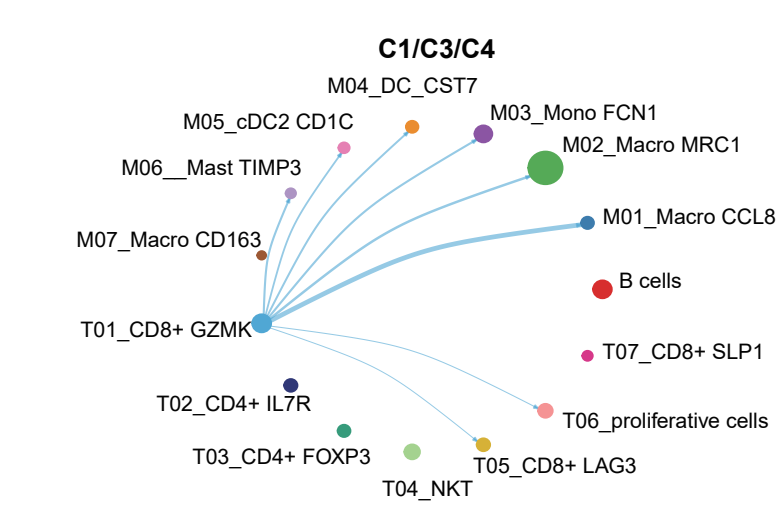

Supplement: Supplementary file 5 — Figure S4 [file 41416_2024_2894_MOESM5_ESM.pdf]

**A**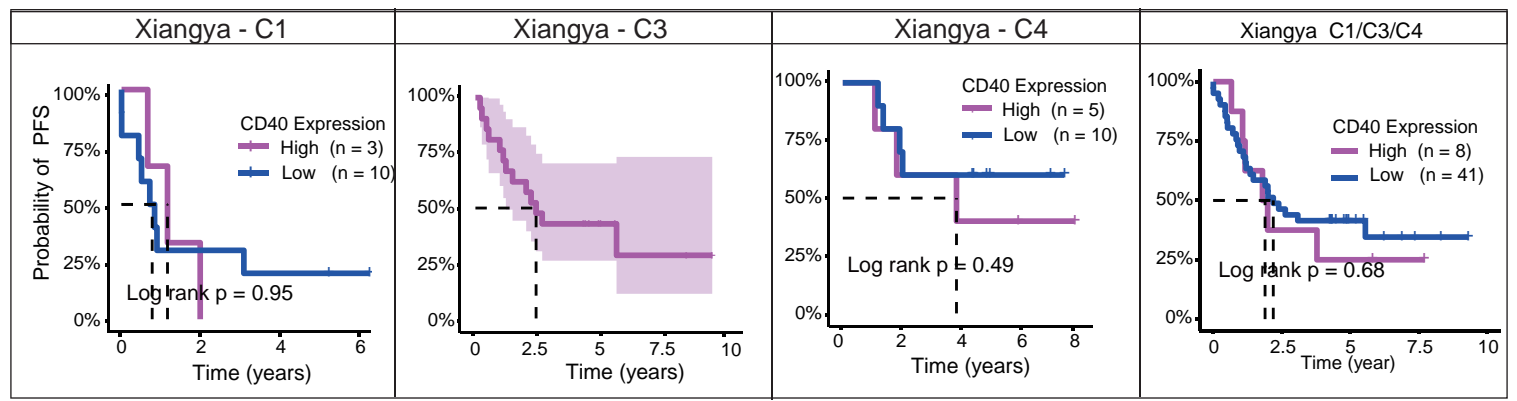**B**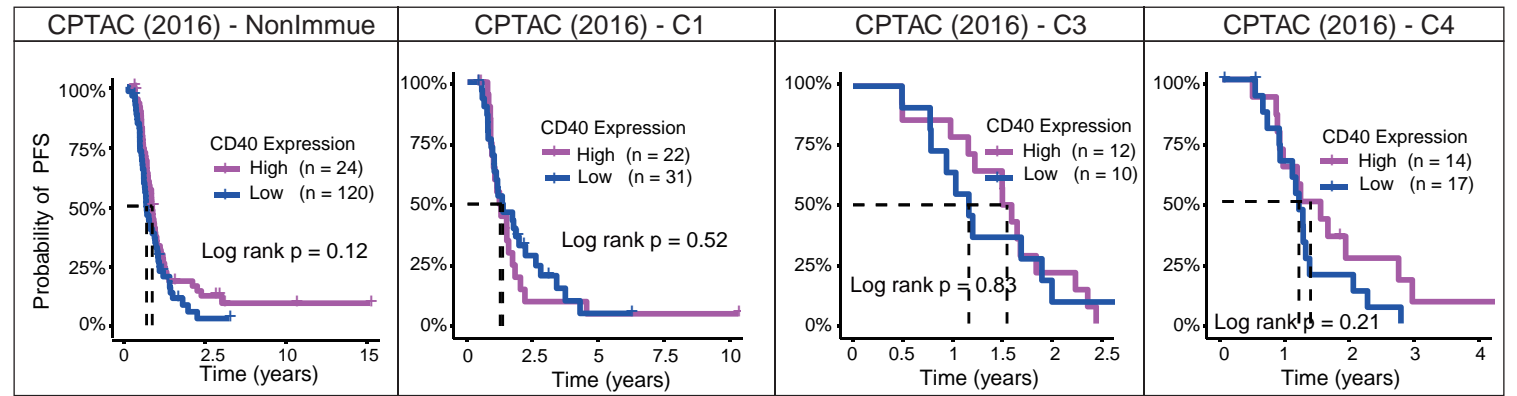**C**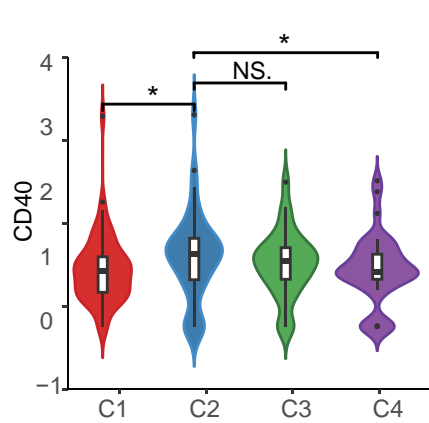**D**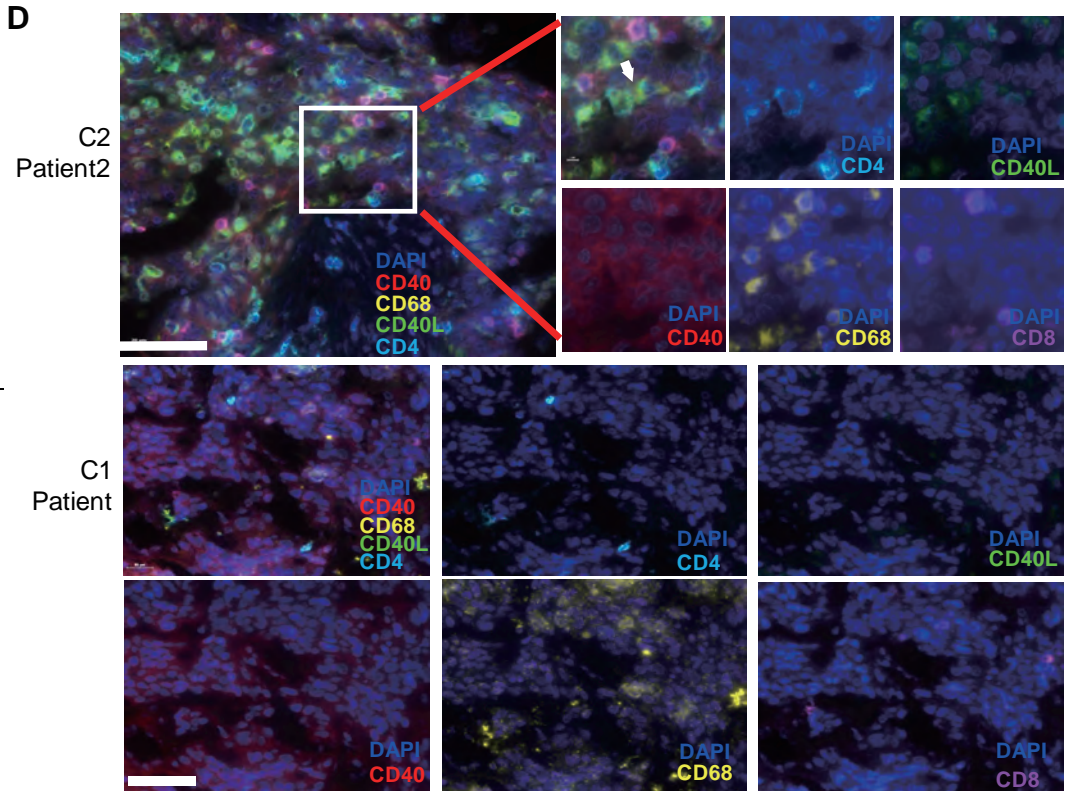

Supplement: Supplementary file 6 — Figure S5 [file 41416_2024_2894_MOESM6_ESM.pdf]

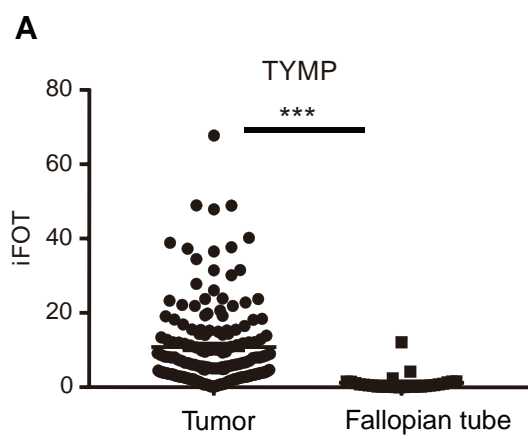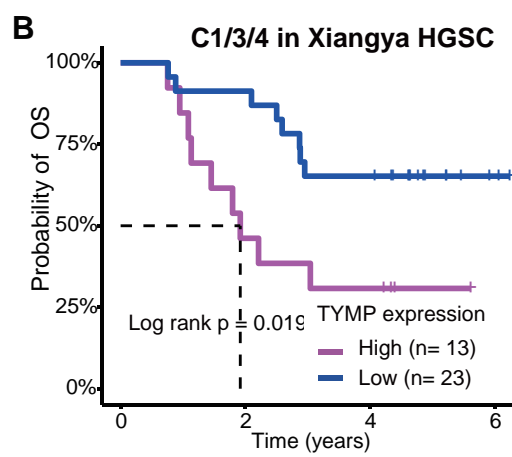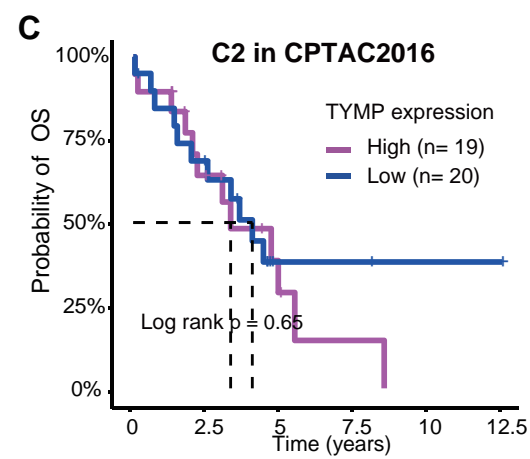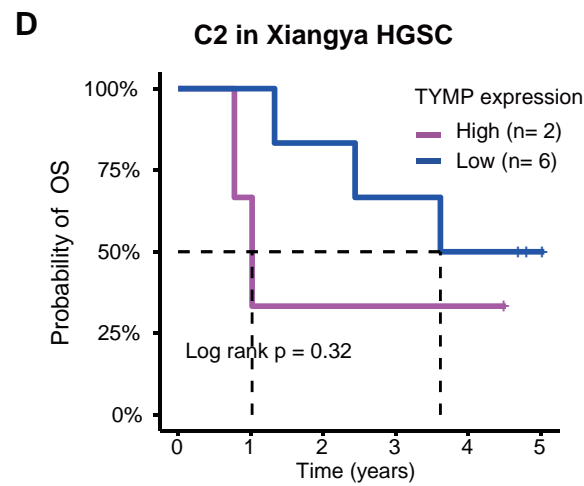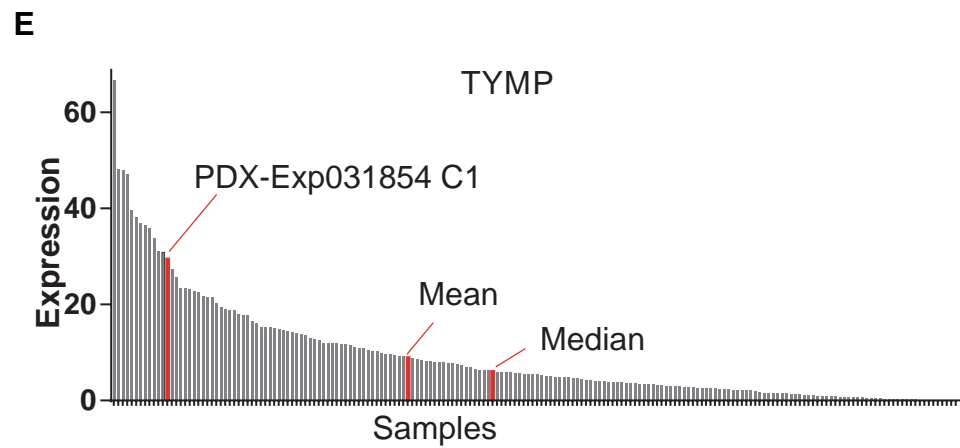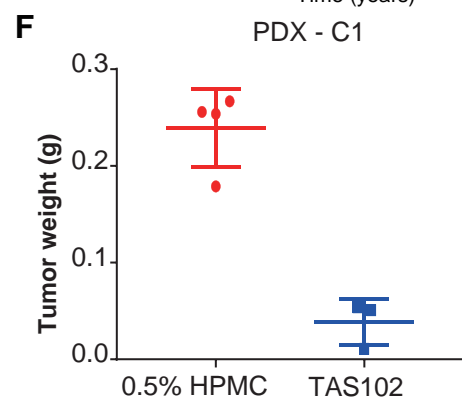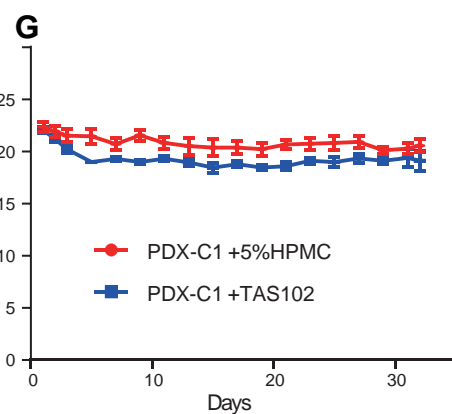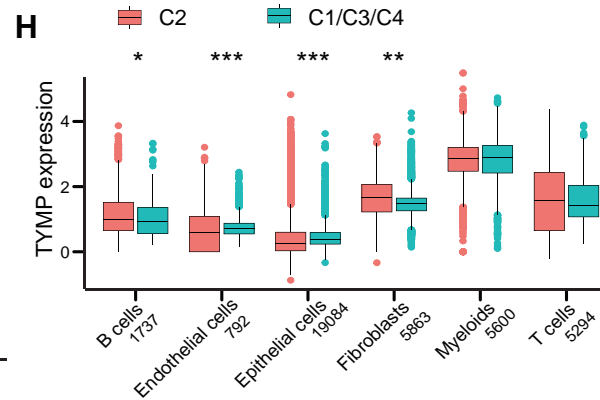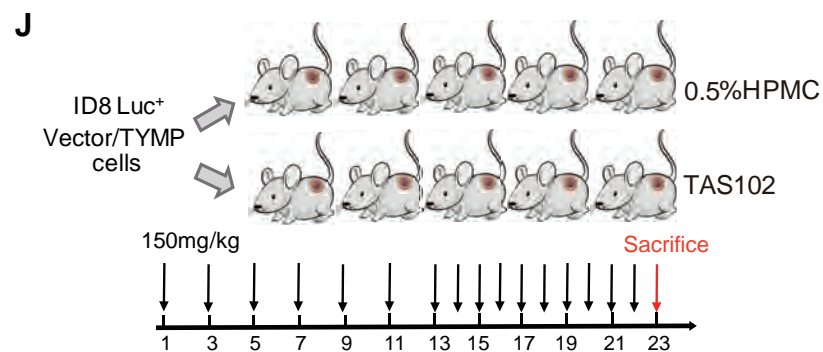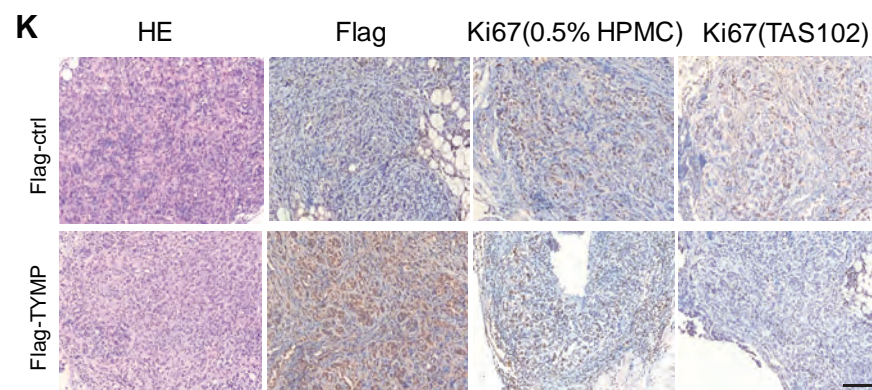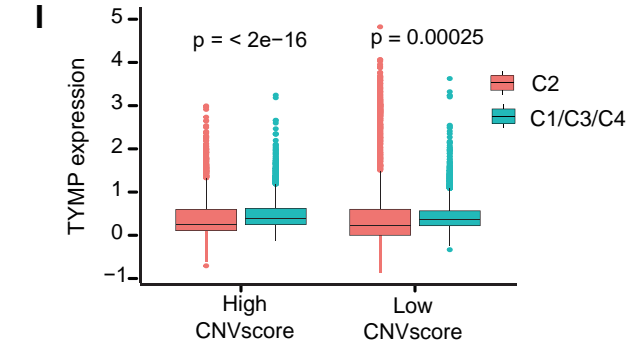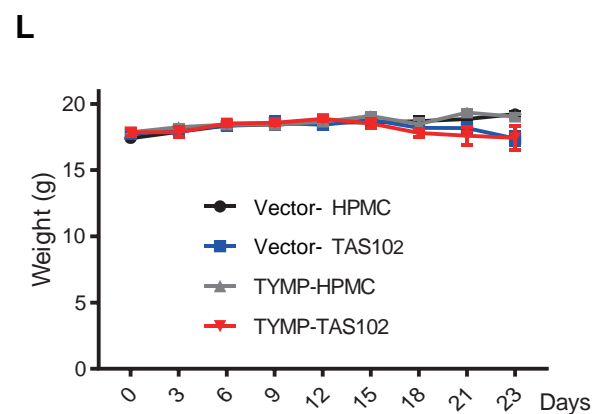

Supplement: Supplementary file 7 — Figure S6 [file 41416_2024_2894_MOESM7_ESM.pdf]
